# Blockade of Activin Receptor IIB Protects Arthritis Pathogenesis by Non‐Amplification of Activin A‐ACVR2B‐NOX4 Axis Pathway

**DOI:** 10.1002/advs.202205161

**Published:** 2023-03-22

**Authors:** Jimin Jeon, Hyemi Lee, Min‐Seung Jeon, Seok‐Jung Kim, Cham Choi, Ki Woo Kim, Dong Joo Yang, Sangho Lee, Yong‐Soo Bae, Won Il Choi, Juyeon Jung, Seong‐il Eyun, Siyoung Yang

**Affiliations:** ^1^ Department of Biological Sciences Sungkyunkwan University Suwon 16419 Republic of Korea; ^2^ CIRNO Sungkyunkwan University Suwon 16419 Republic of Korea; ^3^ Department of Life Science Chung‐Ang University Seoul 06974 Republic of Korea; ^4^ Department of Orthopaedic Surgery Uijeongbu St. Mary's Hospital The Catholic University of Korea College of Medicine Uijeongbu 11765 Republic of Korea; ^5^ MicroCT Applications 3rd floor, 11, Sumyeong‐ro 1‐gil, Gangseo‐gu Seoul 07644 Republic of Korea; ^6^ Department of Oral Biology Yonsei University College of Dentistry Seoul 03722 Republic of Korea; ^7^ Department of Applied Biological Science BK21 FOUR Yonsei University College of Dentistry Seoul 03722 Republic of Korea; ^8^ Center for Bio‐Healthcare Materials Bio‐Convergence Materials R&D Division Korea Institute of Ceramic Engineering and Technology Cheongju Chungbuk 28160 Republic of Korea; ^9^ Bionanotechnology Research Center Korea Research Institute of Bioscience and Biotechnology (KRIBB) Daejeon 34141 Republic of Korea

**Keywords:** ACVR2B assembly, arthritis treatment, druggable target, human OA cartilage, mouse model

## Abstract

Although activin receptor IIB (ACVR2B) is emerging as a novel pathogenic receptor, its ligand and assembled components (or assembly) are totally unknown in the context of osteoarthritis (OA) pathogenesis. The present results suggest that upregulation of ACVR2B and its assembly could affect osteoarthritic cartilage destruction. It is shown that the ACVR2B ligand, activin A, regulates catabolic factor expression through ACVR2B in OA development. Activin A Tg mice (Col2a1‐*Inhba*) exhibit enhanced cartilage destruction, whereas heterozygous activin A KO mice (*Inhba^+/−^*) show protection from cartilage destruction. In silico analysis suggests that the Activin A‐ACVR2B axis is involved in Nox4‐dependent ROS production. Activin A Tg:Nox4 KO (Col2a1‐*Inhba:Nox4^−/−^*) mice show inhibition of experimental OA pathogenesis. NOX4 directly binds to the C‐terminal binding site on ACVR2B‐ACVR1B and amplifies the pathogenic signal for cartilage destruction through SMAD2/3 signaling. Together, the findings reveal that the ACVR2B assembly, which comprises Activin A, ACVR2B, ACVR1B, Nox4, and AP‐1‐induced HIF‐2*α*, accelerates OA development. Furthermore, it is shown that shRNA‐mediated ACVR2B knockdown or trapping ligands of ACVR2B abrogate OA development by competitively disrupting the ACVR2B‐Activin A interaction. These results suggest that the ACVR2B assembly is required to amplify osteoarthritic cartilage destruction and could be a potential therapeutic target in efforts to treat OA.

## Graphical abstract

The blockade of activin receptor IIB can be an effective therapeutic approach for treating osteoarthritis (OA). The intra‐articular injection of Ad‐shACVR2B or sACVR2B‐Fc abrogate OA through inhibition of non‐amplification of the activin A‐ACVR2B‐NOX4 axis. The strategy of competitively disrupting the ACVR2B‐activin A interaction can be used to alleviate OA and may be applicable for treating various activin A‐ACVR2B axis‐related diseases.

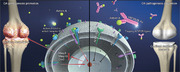

## 1 Introduction

Osteoarthritis (OA) is a leading cause of disability and has a large socioeconomic cost. OA is a whole‐joint disease characterized by cartilage destruction, synovial inflammation, osteophyte formation, subchondral bone sclerosis, and decreased elastic modulus of the cartilage.^[^ ^1^, ^2^ ^]^ However, we currently lack an effective disease‐modifying therapy.^[^ ^3^ ^]^ Among the above‐listed manifestations of OA, cartilage destruction is a hallmark of OA pathogenesis. Consequently, although multiple cell types in joint tissues are known to be involved in OA pathogenesis, chondrocytes have been the focus of most studies on OA pathogenesis. OA cartilage destruction is caused primarily by the upregulation of matrix‐degrading enzymes;^[^ ^4^ ^]^ among them, matrix metalloproteinase 3 (MMP3) and MMP13 are known to play crucial roles.^[^ ^5^, ^6^ ^]^

Cell surface (transmembrane) receptors and their ligands have been the primary therapeutic targets in efforts to combat OA, given their ability to transduce signals through pathogenic receptors.^[^ ^7^, ^8^ ^]^ OA is initiated by several stimulating factors, including pro‐inflammatory cytokines such as interleukin (IL)‐1*β* and IL‐17.^[^ ^9^, ^10^, ^11^ ^]^ These OA‐initiating pathogenic factors, which can be produced by mechanical stress,^[^ ^12^ ^]^ metabolic stress,^[^ ^13^ ^]^ and/or inflammaging,^[^ ^14^ ^]^ bind to their cognate receptors to activate catabolic signaling in chondrocytes.^[^ ^7^ ^]^ This alters biochemical pathways in chondrocytes, leading to extracellular matrix degradation and inflammation through the expression of MMPs and cyclooxygenase‐2 (COX‐2).^[^ ^15^ ^]^ The functional blockade of pathogenic factors and their receptor signaling can be an effective therapeutic approach for treating OA.^[^ ^8^ ^]^

A number of drugs have recently been developed to target these pathways, including several classes of cytokine receptor antagonists, small anti‐inflammatory molecules, and targeted inhibitors of catabolic factors.^[^ ^16^, ^17^ ^]^ Among them, cytokine receptor antagonists and receptor‐trapping ligands are pharmaceutically attractive targets for disrupting OA initiation and progression. Ligand trapping, which is a complementary approach to antibody‐based methods for blocking pathological levels of ligands, offers several advantages.^[^ ^18^ ^]^ In general, a ligand trap is smaller than an antibody, has excellent tissue permeability, and can bind to multiple ligands. A ligand trap will recognize the biologically active part of the target. Given that ligand traps generally have low immunogenicity,^[^ ^19^ ^]^ they do not require protection from the immune system. The joints that are commonly affected by OA are well‐suited to intra‐articular (IA) therapies,^[^ ^20^ ^]^ and using IA injection of a ligand trap for receptor blockade could potentially be developed as a therapeutic approach for OA. IA therapies have a number of physiological and practical advantages over systemic medications, including safety, and IA injection of a relevant ligand trap could offer a novel mechanism of action that more directly targets the pathophysiology of OA.^[^ ^21^ ^]^ However, with the exception of reports on IL‐1 receptor antagonist (IL‐1Ra),^[^ ^22^, ^23^ ^]^ little information is available regarding the potential of using pathogenic receptors as therapeutic targets. Thus, it is important to identify and characterize pathogenic receptors and their ligands to support the development of effective therapeutic targets for treating OA.

ACVR2B is a single‐transmembrane‐domain serine/threonine kinase receptor that acts as a type II receptor; it initiates signaling and cellular responses through binding to ligands, such as GDF5, 8, 11, and activin A.^[^ ^24^, ^25^ ^]^ The downstream function depends on which binding partner engages the receptor.^[^ ^7^ ^]^ Once a ligand is bound to a type II receptor (ACVR2A, ACVR2B, or BMPR2), the type II receptor phosphorylates a type I receptor (ACVR1A, ACVR1B, ACVR1C, BMPR1A, or BMPR1B) to activate kinase activity and thereby trigger signaling via the Smad pathway.^[^ ^25^, ^26^ ^]^ In this manner, the various ligands signal through type I and type II receptors that, upon ligand binding, assemble into the final receptor complex (here, called the “assembly”).^[^ ^24^ ^]^ ACVR2B is emerging as a pathogenic receptor, but no published study has clearly explored its involvement in arthritis pathogenesis or the relevant ligand(s), heteromeric partner receptor(s), and/or downstream mediator(s).

In the present study, we used bioinformatic approaches along with in vitro and in vivo analyses to characterize the function of ACVR2B and its assembly in OA pathogenesis. The various ligands signal through type I and type II receptors which, upon ligand binding, assemble the final receptor complex.^[^ ^24^ ^]^ We reveal that the activin A‐ACVR2B‐Nox4 axis in chondrocytes functions as an essential catabolic regulator of OA through the AP‐1 regulated transcription factor, HIF‐2*α*. As if assembly into heteromeric complexes increases downstream signaling cascades,^[^ ^27^ ^]^ ACVR2B heterodimer with ACVR1B and phosphorylates Smad2/3 signaling pathway, and Nox4 works as a modulator of ACVR2B assembly. Finally, we suggest future translational research with trapping ligands of ACVR2B for treating various types of OA. In sum, we herein reveal the ligand and assembly of ACVR2B as being druggable targets for OA treatment, and characterize the relevant functions and underlying mechanisms of the activin A‐ACVR2B‐Nox4 axis in OA pathogenesis.

## 2 Results

### 2.1 Upregulation of ACVR2B in Cartilage Is Necessary for OA Pathogenesis

**Figure 1:**
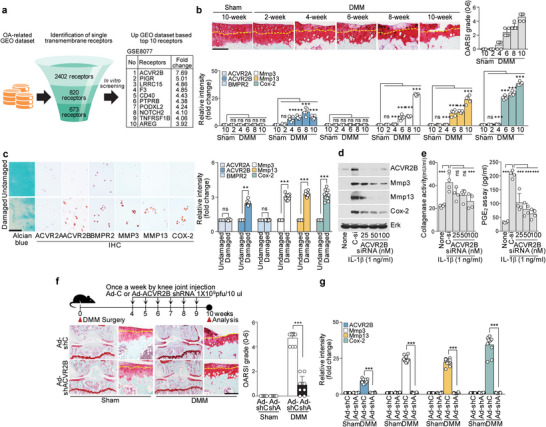
Upregulation of ACVR2B in OA cartilage is necessary for OA pathogenesis. a) Schematic summary of screening for novel pathogenic receptors. b) DMM‐operated mice were sacrificed at the indicated weeks after surgery (*n* = 5). Safranin‐O staining images of cartilage sections (upper left), scoring of OARSI grade (upper right), and immunostaining image density analysis (lower). c) Upregulation of ACVR2B in human OA cartilage (*n* = 10). Images show Alcian blue and immunostaining of ACVR2B in human OA cartilage (left) and analysis of immunostaining intensities (right). d,e) Primary‐culture mouse articular chondrocytes were treated with ACVR2B siRNA (*n* = 4). Shown are (d) Western blot images of the indicated molecules, along with (e) collagenase activity (left) and PGE_2_ production (right). f,g) DMM‐operated WT mice were IA injected with Ad‐ACVR2B shRNA (Ad‐shA) in the joint tissues. Shown are a schematic of the experimental procedures used for Ad‐ACVR2B shRNA injection (f, upper left), Safranin‐O staining images of joint sections (*n* = 10) (f, upper right), scoring of OARSI grade (f, right), and IHC intensity analysis (g). Yellow dotted lines indicate tidemarks (b,f). Values are presented as means ± SD, and were assessed using two‐tailed *t*‐test (c; right), one‐way ANOVA with Bonferroni's post‐hoc test (b, e, g), and Kruskal‐Wallis test followed by Mann‐Whitney U test (f). **p < 0.01; ***p < 0.001, ns; not significant. Scale bar: 100 µm. Ad‐shC, control shRNA adenovirus. Ad‐shA, ACVR2B shRNA adenovirus.

Many pharmaceutical companies have targeted numerous single‐pass transmembrane (TM) receptors as potential therapeutic targets because it is relatively simple to predict and purify their active sites for interactions with pathogenic ligands, such as IL‐1*β* and IL‐17.^[^ ^10^, ^11^ ^]^ To identify new single transmembrane pathogenic receptors involved in OA development, we screened 2402 transmembrane receptors obtained from the GEO database of human OA (GSE16464) and rat OA samples (GSE8077). First, the 2402 transmembrane receptors were sorted by IPA; from those results, we excluded 1582 G‐protein‐coupled receptors (GPCRs). From among the remaining 820 transmembrane receptors, single or multiple transmembrane receptors were identified using UniProtKB/Swiss‐Prot analysis. This resulted in the selection of 673 candidates. From a list of single transmembrane receptors, we excluded known functional receptors and screened the remaining candidates for expression in primary cultured chondrocytes under pathogenic conditions (e.g., in the presence of IL‐1*β*). On the basis of our results, we identified ACVR2B as a possible pathogenic single‐pass TM receptor involved in OA pathogenesis (**Figure** **1**a; and Table S1, Supporting Information). ACVR2B is a TM serine/threonine kinase receptor that is known to be involved in many physiological and pathological processes.^[^ ^12^ ^]^ However, its function in OA pathogenesis has not previously been assessed. We therefore examined the possible functions and underlying regulatory mechanisms of ACVR2B in OA pathogenesis.

As ACVR2B is a type II receptor, we further assessed the possible involvement of two other type II receptors, ACVR2A and BMPR2, in OA pathogenesis. Mouse OA caused by DMM (destabilization of the medial meniscus) surgery provides a reproducible and slow‐progressing disease that resembles the development of human OA.^[^ ^28^ ^]^ In this OA mouse model, ACVR2B began to increase gradually at 4 weeks post‐surgery, before changes were seen in the expression levels of MMP3, MMP13, and COX‐2; in contrast, the levels of ACVR2A and BMPR2 were unchanged throughout the observation period (Figure 1b; and Figure S1a,b, Supporting Information). Moreover, the protein levels of ACVR2B (but not ACVR2A or BMPR2) were markedly elevated in OA‐affected, damaged regions of human cartilage compared with undamaged areas from the same patient (Figure 1c). These findings suggest that ACVR2B could be related to OA development. The OA cartilage also exhibited elevated levels of MMP3 and MMP13, which play crucial roles in OA cartilage destruction,^[^ ^5^, ^6^ ^]^ and COX‐2, which is a key enzyme for prostaglandin E2 (PGE_2_) production and inflammatory responses.^[^ ^15^ ^]^ To characterize the function of ACVR2B in chondrocytes, we performed ACVR2B loss‐of‐function experiments in vitro and in vivo under OA‐mimicking conditions. The siRNA‐mediated knockdown of ACVR2B in primary‐culture chondrocytes inhibited the abilities of IL‐1*β* to upregulate the productions of MMPs, COX‐2, and PGE_2_ and the activity of collagenase (Figure 1d,e), suggesting that ACVR2B is involved in the pro‐inflammatory cytokine‐induced expression of these OA mediators in chondrocytes. The in vivo function of ACVR2B was addressed by its knockdown in joint tissues with intra‐articular (IA) injection of an adenovirus expressing an shRNA against ACVR2B (Ad‐shACVR2B). The knockdown of ACVR2B significantly abrogated DMM‐induced cartilage destruction and catabolic factor expression (Figure 1f,g; and Figure S1c, Supporting Information).

### 2.2 The Activin A‐ACVR2B Axis Works as a Critical Catabolic Inducer of OA Development

**Figure 2:**
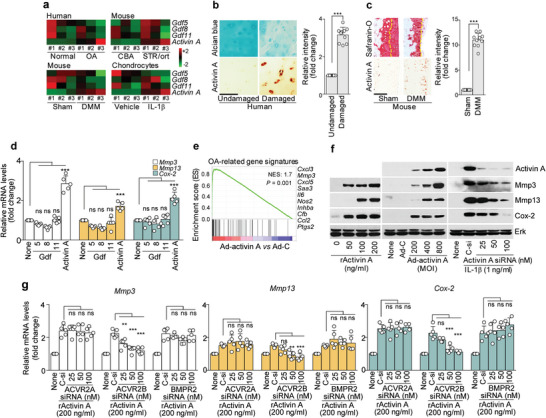
Activin A is associated with OA through ACVR2B. a) Heatmap of Gdf5, Gdf8, Gdf11, and activin A expression in cartilage from human OA patients, SRT/ort OA mice, and DMM‐operated mice, and in IL‐1*β* treated human chondrocytes. b,c) Images of Alcian blue or Safranin‐O staining and activin A immunostaining in human OA cartilage and DMM‐operated mouse cartilage (b,c; left, *n* = 10), with immunostaining intensity (b,c; right). d) The indicated molecules were determined by qRT‐PCR analysis of mouse primary chondrocytes treated with 200 ng mL^−1^ of Gdf5, 8, 11, or activin A. e) GSEA of OA signature genes in chondrocytes infected with Ad‐C or Ad‐activin A. f) Western blot images of the indicated molecules after chondrocytes were treated with recombinant activin A (left), infected with Ad‐activin A (middle), or treated with the indicated concentrations of activin A siRNA (right). g) *Mmp3, Mmp13*, and *Cox‐2* expression levels after knockdown of ACVR2A, ACVR2B, or BMPR2 in activin A‐treated chondrocytes. Tidemarks are indicated by yellow dotted lines (c). Values are presented as means ± SD, and were assessed using two‐tailed *t*‐test (b,c) or one‐way ANOVA with Bonferroni's post‐hoc test (d,g). **p < 0.01; ***p < 0.001.

We next characterized ligands of ACVR2B that appear to be associated with OA pathogenesis. Regarding upstream activators, growth differentiation factor (GDF)5, 8, 11, and activin A (encoded by *Inhba*) are known to be ligands for ACVR2B.^[^ ^24^ ^]^ To identify the ligands of ACVR2B in OA pathogenesis, we performed in silico analysis against human and mouse OA GEO databases (GSE16464, GSE33754, GSE26475, and GSE75181). Activin A (but not the other known ligands) was markedly upregulated in human OA cartilage, DMM‐induced mouse OA cartilage, OA cartilage of STR/ort mice (a spontaneous OA model),^[^ ^29^ ^]^ and primary‐culture chondrocytes treated with IL‐1*β* or TNF‐*α* (**Figure** **2**a; and Figure S2a, Supporting Information). Similar results were observed in damaged regions of human cartilage and DMM induced‐OA mouse cartilage (Figure 2b,c). IHC analyses demonstrated that activin A was significantly upregulated at 4 weeks post‐surgery, before the observed cartilage destruction and catabolic factor expression, whereas no such change was seen for GDF5, 8, or 11 (Figure S2b,c, Supporting Information). We next assessed whether ACVR2B ligands were involved in OA pathogenesis. Treatment of chondrocytes with recombinant versions of the ACVR2B ligands, GDF5, GDF8, GDF11, or activin A, revealed that GDF5 induced anabolic factor expression, activin A regulated catabolic factor expression, and GDF8 and 11 did not affect anabolic or catabolic factor expression (Figure 2d; and Figure S2d, Supporting Information). As previous research suggested that activin A functions as an autocrine regulator,^[^ ^30^ ^]^ we also assessed whether activin A was expressed in GDF5, GDF8, GDF11, or activin A‐treated chondrocytes. Indeed, activin A was further increased in activin A‐treated chondrocytes, but not in those treated with GDF5, GDF8, or GDF11 (Figure S2e, SupportingInformation). This result suggested that activin A could act as an autocrine regulator in OA pathogenesis. Gene set enrichment analysis (GSEA) further suggested that the OA‐signature gene set was positively enriched for activin A, and micromass culture analysis revealed that activin A did not affect chondrogenesis (Figure 2e; and Figure S3a–c, Supporting Information). We identified a specific activin A gene signature and found that it did not overlap with the signatures seen in chondrocytes treated with GDF5, 8, or 11, suggesting that the activin A signature may reflect genes that are involved in OA pathogenesis (Table S2, Supporting Information). To characterize the function of activin A in OA pathogenesis, we performed gain‐of‐function and loss‐of‐function analyses. Our results demonstrated that overexpression of activin A via recombinant activin A or Ad‐activin A infection triggered the productions of MMP3, MMP13, and COX‐2. Conversely, the siRNA‐mediated knockdown of activin A inhibited the IL‐1*β*‐induced upregulations of MMPs and COX‐2 in chondrocytes (Figure 2f). Moreover, our in vitro knockdown analysis strongly suggested that activin A induces catabolic factor expression through ACVR2B, but not other type II receptors, such as ACVR2A or BMPR2 (Figure 2g).

Together, these findings indicate that activin A stimulates catabolic factor expression through ACVR2B, which is key pathogenic receptor in activin A‐induced OA pathogenesis.

### 2.3 Osteoarthritic Cartilage Destruction Reflects the Expression of Activin A

**Figure 3:**
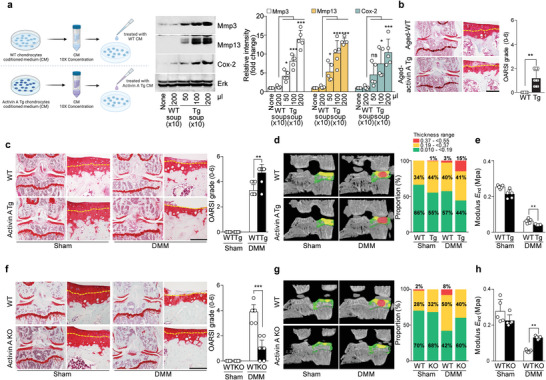
Activin A is a critical catabolic regulator of OA pathogenesis. a) Schematic illustration of the strategy used to concentrate conditioned media from chondrocytes of WT and activin A Tg mice (left), along with Western blot images (middle) and relative protein intensity levels (right) of the indicated molecules. The culture medium from Wt and activin A Tg chondrocytes were collected for 24 h, concentrated it to 10×, and applied the indicated volumes (in µL) of the concentrate to normal chondrocytes. (*n* = 5). b) Cartilage destruction in 18 month old WT (*n* = 5) and activin A Tg (*n* = 9) mice was determined by Safranin‐O staining (left) and OARSI scoring (right). c) Safranin‐O staining images of joint sections (left) and scoring of OARSI grade (right) (*n* = 10). d) 3D µCT images (left; *n* = 5) and stacked‐bar plot showing the trabecular bone thickness distribution of the indicated samples (right). e) The elastic modulus of cartilage, as measured by bioindentation (*n* = 5). f) Safranin‐O staining images of joint sections (left) and scoring of OARSI grade (right) (*n* = 10). g) Representative 3D µCT images (left; *n* = 5) and stacked‐bar plot showing the trabecular bone thickness distribution of the indicated samples (right). h) The elastic modulus of cartilage, as measured by bioindentation (*n* = 5). Tidemarks are indicated by yellow dotted lines (b,c,f). Values are presented as means ± SD and were assessed using one‐way ANOVA with Bonferroni's post‐hoc test (a), two‐tailed *t*‐test (b), or Kruskal‐Wallis test followed by Mann‐Whitney U test (c,e,f,h). **p < 0.01; ***p < 0.001. Scale bar: 100 µm.

We further evaluated the catabolic role of activin A by generating cartilage‐specific activin A transgenic (Tg) mice (Col2a1‐*Inhba*) using the Col2a1 promoter and enhancer.^[^ ^31^ ^]^ We first tested activin A expression in activin A Tg mice, and then assessed parameters of OA pathogenesis in vitro and in vivo. The chondrocytes of postnatal activin A Tg mice showed increased expression levels of catabolic factors (MMP3, MMP13, and COX‐2) compared to those of WT mice (Figure S4a,b, Supporting Information). We found that activin A was secreted to the culture media of chondrocytes obtained from postnatal activin A Tg mice. A 10× concentration of culture medium from chondrocytes of postnatal activin A Tg mice applied at various doses affected the expression levels of catabolic factors in WT chondrocytes (**Figure** **3**a). As observed in vitro, activin A Tg mice spontaneously exhibited OA pathogenesis and catabolic factor expression (Figure 3b; and Figure S4c, Supporting Information). Interestingly, DMM‐induced activin A Tg mice exhibited accelerated osteoarthritic cartilage destruction and catabolic factor expression, compared to DMM‐induced WT mice (Figure 3c; and Figure S5a, Supporting Information). Furthermore, µCT analysis suggested that the pathological alterations of subchondral bone were increased in DMM‐induced activin A Tg mice compared to the corresponding WT mice (Figure 3d). To provide information on critical mechanical and compositional cartilage traits, we analyzed the elastic modulus of the cartilage using the bioindentation technique. The modulus of elasticity results indicated that activin A Tg cartilage was severely damaged compared to WT mouse cartilage (Figure 3e). We next used heterozygous activin A knockout (KO) mice [*Inhba* ^+/−^, chosen because *Inhba* ^−/−^ mice are embryonic lethal^[^ ^32^ ^]^] to assess whether heterozygous activin A KO mice could protect against OA cartilage destruction. In DMM‐operated heterozygous activin A KO mice, we observed suppression of osteoarthritic cartilage destruction and catabolic factor expression, compared to the same parameters in DMM‐induced WT mice (Figure 3f; and Figure S5b, Supporting Information). µCT and elastic modulus of the cartilage analyses revealed that depletion of activin A could protect against OA pathogenesis and rescue the elastic modulus of cartilage tissue (Figure 3g,h). Taken together, these results strongly indicate that activin A could critically accelerate OA pathogenesis through an interaction with ACVR2B.

### 2.4 NADPH Oxidase 4 (Nox4) Is a Critical Catabolic Mediator of the Activin A‐ACVR2B Axis

**Figure 4:**
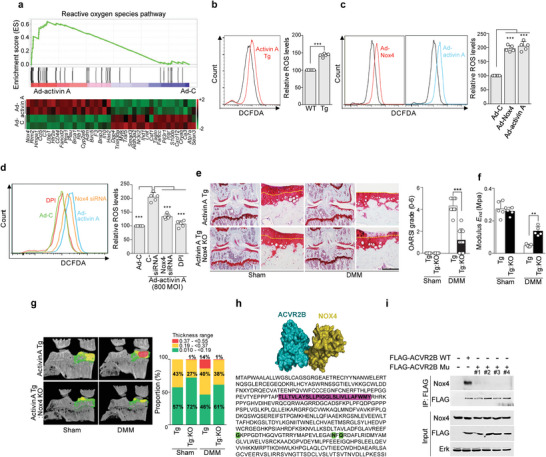
Nox4 is a critical downstream catabolic mediator of the activin A‐ACVR2B axis in OA pathogenesis. a) GSEA (upper) and heatmap of ROS‐related genes (lower) altered following Ad‐activin A infection in chondrocytes. b) Intracellular ROS levels in postnatal chondrocytes of activin A Tg (red line) and WT littermate (black line) mice. c) Intracellular ROS levels in chondrocytes infected with 800 MOI of Ad‐C (black line), Ad‐activin A (blue line), or Ad‐Nox4 (red line). d) Intracellular ROS levels of chondrocytes infected with Ad‐C (orange line) or Ad‐activin A (blue line) in the presence of diphenyleneiodonium (green line) or Nox4 siRNA (yellow line). Quantification of ROS fluorescence intensity (b,c,d; right; *n* = 5). e–g) DMM‐operated activin A Tg:Nox4 KO mice were analyzed. Shown are Safranin‐O staining images of joint sections (e; *n* = 10), the elastic modulus of cartilage determined by bioindentation (f; *n* = 5), 3D µCT images (g; left), and the trabecular bone thickness distribution (g; right; *n* = 5). h) Computational docking models for ACVR2B (cyan) and NOX4 (olive). Pink: ACVR2B transmembrane domain. Green: NOX4 binding sites (*lower*). i) Chondrocytes were transfected with WT ACVR2B or muACVR2B. Cell lysates were subjected to immunoprecipitation (IP) with FLAG (*n* = 3). Yellow dotted lines indicate tidemarks (e). Values are presented as mean ± SD and were analyzed using two‐tailed *t*‐test (b), one‐way ANOVA with Bonferroni's post‐hoc test (c,d), or Kruskal‐Wallis test followed by Mann‐Whitney U test (e,f). **p < 0.01; ***p < 0.001. Scale bar: 100 µm.

Next, we performed GSEA and IPA in Ad‐activin A‐infected chondrocytes to identify downstream mediators of the activin A‐ACVR2B axis in OA pathogenesis. GSEA and IPA revealed that most of the genes upregulated in Ad‐activin A‐infected chondrocytes were related to reactive oxygen species (ROS) production (**Figure** **4**a, upper; and Figure S6a, Supporting Information). Microarray analysis identified Nox4 as the most highly upregulated gene in activin A‐overexpressing chondrocytes (Figure 4a, lower). NOX4 is known to be responsible for ROS production in many cell types.^[^ ^33^ ^]^ We monitored ROS production with DCF‐DA staining analysis and assessment of 8‐hydroxy 2 deoxyguanosine (8‐OHdG, a major product of ROS damage) for in vitro and in vivo conditions, respectively.^[^ ^34^ ^]^ Consistently, Nox4 expression and ROS production were upregulated in activin A‐overexpressed chondrocytes and spontaneous activin A Tg mice (Figure 4b; and Figure S6b,c, Supporting Information), whereas knockdown of Nox4 inhibited activin A‐induced ROS production (Figure 4c,d). As NOX4 is known to contribute to OA cartilage destruction through inflammation induction and matrix degradation,^[^ ^35^ ^]^ we next assessed OA cartilage destruction in DMM‐induced Nox4 KO mice. Our results first revealed that Nox4 KO mice exhibited significant abrogation of osteoarthritic cartilage destruction and catabolic factor expression (Figure S6d,e, Supporting Information). We found that Nox4 overexpression in chondrocytes upregulated MMP3, MMP13, and COX‐2, whereas siRNA‐mediated knockdown of Nox4 and ACVR2B abrogated the activin A‐induced upregulations of these catabolic factors (Figure S6f–h, Supporting Information). We further observed rescue of pathological alterations in subchondral bone and the elastic modulus of the cartilage tissue in DMM‐induced Nox4 KO mice, as assessed by µCT and bioindentation analyses, respectively (Figure S6i,j, Supporting Information). To assess the in vivo function of NOX4 as a mediator of the activin A‐ACVR2B axis, we generated activin A Tg:Nox4 KO (Col2a1‐*Inhba*:*Nox4* ^−/−^) mice by crossing cartilage‐specific activin A Tg mice with Nox4 KO mice. The enhancements of OA manifestations, catabolic mediators, and ROS production seen in DMM‐operated activin A Tg mice were significantly inhibited by knockout of Nox4 in activin A Tg:Nox4 KO mice (Figure 4e–g; and Figure S7, Supporting Information). Interestingly, we found that a direct interaction between ACVR2B and NOX4 was supported by our in silico protein structural homology modeling (Figure 4h). To examine whether Nox4 is a direct downstream mediator of the activin A‐ACVR2B axis, we performed co‐immunoprecipitation (co‐IP) analysis with ACVR2B in activin A‐overexpression conditions. NOX4 consists of six transmembrane (TM) domains: Its N‐ and C‐terminal moieties both face the cytosol^[^ ^36^ ^]^ and its C‐terminus could interact with a C‐terminal binding site on ACVR2B. NOX4 is predicted to bind to the C‐terminus‐proximal residues, G373, N399, and Q401, of ACVR2B (Figure 4h, lower). We performed co‐IP analyses with WT ACVR2B (wtACVR2B) and mutated forms of ACVR2B (Mu1, with G373 changed to K373; Mu2, with G399 changed to K399; Mu3, with Q401 changed to K401; and Mu4, with G373, G399, and Q401 changed to K373, K399, and K401, respectively). Our co‐IP analyses indicated that endogenous Nox4 bound to the exogenous wtACVR2B sequences, but not the exogenous muACVR2B sequences (Figure 4i). Taken together, these findings indicate that the C‐terminal sequence of ACVR2B directly interacts with Nox4 and further suggest that Nox4 could act as a downstream mediator of the signaling pathway for OA pathogenesis.

### 2.5 ACVR1B and AP‐1 Are Required to Complete the ACVR2B Assembly and Thereby Accelerate OA

Once activin A binds to ACVR2B, the latter should recruit a type I receptor (ACVR1A, ACVR1B, ACVR1C, BMPR1A, or BMPR1B) to trigger kinase activity for Smad signaling.^[^ ^37^, ^38^ ^]^ Here, we found that pSmad2/3, but not pSmad1/5 or non‐Smad signaling, is involved in the activin A‐ACVR2B‐Nox4 axis and subsequent OA pathogenesis (Figure S8a–e, Supporting Information). Furthermore, Activin A Tg and activin A Tg:Nox4^+/−^ heterozygous chondrocytes showed Smad2/3phosphorylation increased relative to controls, whereas activin A Tg:Nox4 KO^−/−^ and Nox4 null chondrocytes exhibited reduced Smad2/3 phosphorylation (Figure S8f, Supporting Information). These data are exactly concordant with our in vitro data; together, they collectively suggest that Nox4 could amplify Smad2/3 phosphorylation in the activin A‐ACVR2B‐Nox4 axis. Based on the well‐known functions of BMPs in chondrogenesis,^[^ ^39^ ^]^ we speculated that BMP‐Smad1/5/9 signaling could also be upregulated to contribute to the OA phenotype. To check whether BMPs function through ACVR2B in chondrocytes, we performed in vitro analysis with rBMP2, 4, and 7‐treated chondrocytes in the absence or presence of ACVR2B siRNA. We found that BMP2, 4, and 7 failed to regulate catabolic factor expression (Figure S8g, Supporting Information).

**Figure 5:**
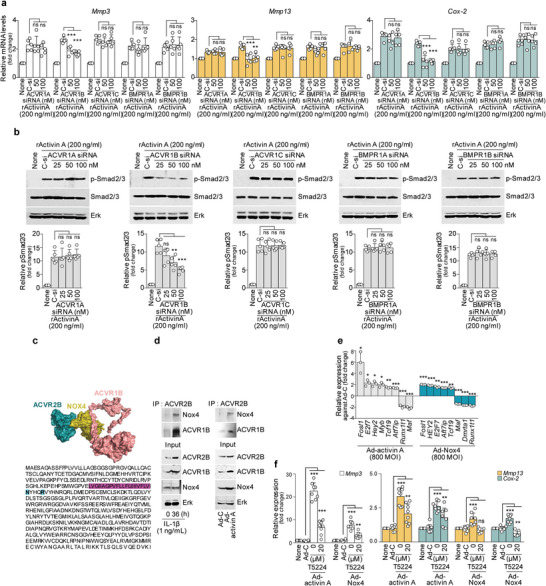
ACVR1B and AP‐1 are required to complete the ACVR2B assembly and thereby accelerate OA. a,b) Chondrocytes were treated with rActivin A (200 ng mL^−1^) in the absence or presence of 100 × 10^−9^ m of control siRNA (C‐si) or 50 × 10^−9^ m to 100 × 10^−9^ m of siRNA against type I receptors (ACVR1A, ACVR1B, ACVR1C, BMPR1A, or BMPR1B; *n* = 5). a) Relative mRNA levels of the indicated molecules, as assessed by qRT‐PCR analysis. b) Representative Western blot images (upper) and relative protein intensity levels (lower) of p‐Smad2/3 in type I receptor‐knockdown chondrocytes treated with rActivin A. c) Computational docking models for ACVR2B (cyan), ACVR1B (pink), and NOX4 (olive) (upper). Purple: ACVR1B transmembrane domain. Green: NOX4 binding sites (lower). d) Interaction of endogenous ACVR2B and ACVR1B heterodimers with endogenous Nox4 in IL‐1*β*‐treated or Ad‐activin A‐infected chondrocytes (*n* = 3). e) Profiling of activin A‐ or Nox4‐induced transcription factors. List of the highest‐ and lowest‐expressed transcription factors in Ad‐activin A‐ or Ad‐Nox4‐infected chondrocytes. Ad‐C‐infected chondrocytes were used as controls. f) Chondrocytes were infected with Ad‐C, Ad‐activin A, or Ad‐Nox4 in the presence of 20 × 10^−6^ m of T5224 for 24 h. Representative qRT‐PCR analysis results for the indicated molecules (*n* = 8). Values are presented as means ± SD and were assessed using one‐way ANOVA with Bonferroni's post‐hoc test (a,b,f) and two‐tailed *t*‐test (e). *p < 0.05; **p < 0.01; ***p < 0.001, ns; not significant.

To next clearly characterize the involvement of type I receptors in activin A‐induced catabolic factor expression, we performed siRNA analysis of all type I receptors. The results indicated that activin A regulates catabolic factor expression through Smad2/3 phosphorylation via ACVR1B, but not the other type I receptors (**Figure** **5**a,b). We additionally used two docking programs, AlphaFold2 and ClusPro, and the results demonstrated that Nox4 could bind to the C‐terminus‐proximal residues, 150N and R154, of ACVR1B in the ACVR2B‐ACVR1B heterodimer (Figure 5c) Moreover, when endogenous Nox4 was overexpressed in chondrocytes through IL‐1*β* treatment or Ad‐activin A infection, co‐IP analysis showed that the endogenous Nox4 directly interacted with the endogenous ACVR2B‐ACVR1B heterodimer (Figure 5d). These results indicate that Nox4 modulates the activin A‐ACVR2B/ACVR1B axis and contributes to Smad2/3 signaling‐mediated catabolic factor expression.

We next searched for target transcription factor(s) of the activin A‐ACVR2B‐Nox4 axis by screening a transcription factor IPA library in primary‐culture mouse chondrocytes infected with Ad‐C, Ad‐activin A, or Ad‐Nox4. Among the examined transcription factors, overexpression of activin A or NOX4 most highly upregulated Fosl1 (Fos‐related antigen 1, also known as Fos‐like 1 and encoded by Fosl1), compared to the level seen in Ad‐C‐infected chondrocytes (Figure 5e). Fosl1 is an AP‐1 transcription factor subunit known to activate the Smad signaling pathway.^[^ ^40^ ^]^ Fosl1, Fosl2, Fos, FosB, cJun, JunB, and JunD can multimerize to form the AP‐1 transcription factor complex.^[^ ^41^ ^]^ qRT‐PCR demonstrated that most subunits of AP‐1 were upregulated by overexpression of activin A or Nox4, compared to the corresponding controls (Figure S9a, Supporting Information). T5224 is known as an AP‐1 inhibitor.^[^ ^42^ ^]^ We compared chondrocytes treated with Ad‐activin A or Ad‐Nox4 infection with or without 20 × 10^−6^ m of T5224, and those treated with Ad‐C infection versus Ad‐activin A or Ad‐Nox4 infection with or without 20 × 10^−6^ m of T5224. The results were statically analyzed using one‐way ANOVA with Bonferroni's post‐hoc test. The data collectively suggested that the AP‐1 inhibitor, T5224, significantly attenuated the upregulations of MMP3, MMP13, and COX‐2 in Ad‐activin A‐ or Ad‐Nox4‐infected chondrocytes (Figure 5f). HIF‐2*α*, which is a master transcriptional regulator in OA pathogenesis,^[^ ^31^ ^]^ contains an AP‐1 binding site in its promoter (Figure S9b, Supporting Information). Interestingly, rActivin A treatment and Ad‐activin A infection of chondrocytes increased HIF‐2*α* expression and activity, whereas an AP‐1 inhibitor blocked HIF‐2*α* expression in our system (Figure S9c, Supporting Information). Based on these findings, we propose that AP‐1, which is regulated by the activin A‐ACVR2B‐Nox4 axis, can modulate catabolic factor expression via regulating HIF‐2*α* in OA development. Our findings together reveal that the Fosl1‐mediated AP‐1 transcription factor can mediate the HIF‐2*α* regulated catabolic action of the activin A‐ ACVR2B ‐Nox4 axis and appears to mediate completion/activation of the ACVR2B assembly through Smad2/3 signaling via ACVR1B in OA pathogenesis.

### 2.6 Trapping of the ACVR2B Ligand, Activin A, Abrogates OA Pathogenesis via Competitively Disrupting the ACVR2B–Activin A Interaction

**Figure 6:**
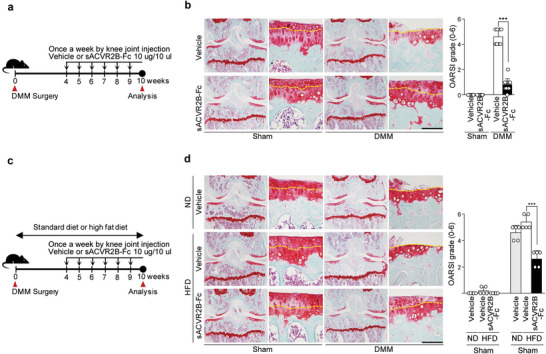
Trapping activin receptor IIB (ACVR2B) ligands attenuates OA and metabolic OA pathogeneses. a,b) Sham‐ or DMM‐operated WT mice were IA injected with PBS as a vehicle or sACVR2B‐Fc (10 µg in a total volume of 10 µL) to block interactions between ACVR2B and its ligands, and sacrificed at 10 weeks after the surgery (*n* = 10). a) Schematic of the experimental procedure used for vehicle or sACVR2B‐Fc knee‐joint injection prior to DMM surgery. b) Representative Safranin‐O staining images of joint sections (left) and scoring of OARSI grade (right). c,d) HFD‐fed sham‐ or DMM‐operated WT mice were sacrificed at the indicated days after surgery (*n* = 5). c) Schematic showing the experimental procedure for a vehicle or sACVR2B‐Fc knee‐joint injection in HFD‐fed mice induced with DMM surgery. d) Representative Safranin‐O staining images of cartilage sections (left) and scoring of OARSI grade (right). Tidemarks are indicated by yellow dotted lines (b,d). Values are presented as means ± SD and were assessed using the Kruskal‐Wallis test followed by Mann‐Whitney U test (b,d; right). *p < 0.05; **p < 0.01; ***p < 0.001, ns; not significant. Scale bar: 100 µm.

Type II receptors are required for ligand binding, and soluble ACVR2B (sACVR2B‐Fc) is known to trap ligands of ACVR2B and block its downstream activation by competitively disrupting the receptor‐ligand interaction.^[^ ^37^, ^38^, ^43^ ^]^ A previous SPR‐based binding assay suggested that ACVR2B binds activin A with higher affinity than seen for another of its ligands.^[^ ^44^ ^]^ To explore the feasibility of targeting ACVR2B for OA therapy, we performed IA injection of sACVR2B‐Fc in various OA mouse models. We found that weekly IA injections of sACVR2B‐Fc over a period of 7 weeks significantly abrogated DMM‐induced osteoarthritic cartilage destruction (**Figure** **6**a,b). Since OA in patients is largely related to metabolic factors, and metabolic changes induced by high‐fat diet (HFD) could contribute to OA,^[^ ^45^ ^]^ we assessed whether trapping a ligand of ACVR2B could protect against HFD‐induced OA in mice, as a representative metabolism‐related OA pathogenesis. Although HFD increased glucose and insulin tolerance in WT mice, HFD alone was not sufficient to induce OA cartilage destruction (Figure S10a–d, Supporting Information). However, a previous report suggested that HFD could accelerate the OA cartilage destruction induced by DMM surgery.^[^ ^45^ ^]^ We tested whether sACVR2B‐Fc could protect against this HFD‐induced OA pathogenesis and found that, indeed, sACVR2B‐Fc protected against OA cartilage destruction in this metabolic‐OA‐mimicking condition (Figure 6c,d). Taken together, these results show that trapping a ligand of ACVR2B could be a useful strategy for blocking ACVR2B assembly activation in OA pathogeneses.

## 3 Discussion

The binding of diverse pathogenic ligands (e.g., proinflammatory cytokines and growth factors) to their specific receptors induces catabolic factor expression in chondrocytes and causes cartilage degradation, eventually promoting OA progression. Cell surface receptors, their ligands, and downstream mediators have been shown to form pathogenic receptor assemblies. Such assemblies have been the focus of significant research efforts aimed at alleviating disease by blocking signals through receptor antagonists or trapping ligands.^[^ ^46^, ^47^ ^]^ For example, IL‐1Ra or monoclonal antibodies against IL‐1*β* were investigated in clinical trials for their ability to inhibit the key OA‐causing cytokine, IL‐1*β*. This treatment was found to inhibit IL‐1 activity and improve OA symptoms within days, but there was no difference in symptoms and an insufficient difference in clinical parameters compared to those of the control group at 1‐month post‐treatment.^[^ ^48^, ^49^ ^]^ Although various clinical trials have been conducted, few drugs are available to inhibit IL‐1 in cartilage, and the molecular mechanisms involved in receptor‐related OA pathogenic processes are still unclear. Here, we report the first in silico identification of ACVR2B as an OA pathogenic frontline receptor. We reveal that in vivo osteoarthritic pathogenic conditions upregulate the expression of ACVR2B, but not other type II receptors (ACVR2A and BMPR2). Although BMPRs share the ACVR2B signaling pathway,^[^ ^50^ ^]^ we demonstrate that BMP2, 4, and 7 fail to regulate catabolic factor expression with or without ACVR2B. We further show that depletion of ACVR2B by IA injection of Ad‐ACVR2B shRNA inhibits the expression levels of MMP3, MMP13, and COX‐2. These results clearly support the idea that the expression and activation of ACVR2B are closely related to OA pathogenesis. To further explore this concept, we identified the pathogenic ligands of ACVR2B in OA pathogenesis. Among the ligands of ACVR2B, GDF5 and 11 play roles in skeletal development and GDF8 enhances bone repair and regeneration.^[^ ^51^, ^52^, ^53^ ^]^ GDF5 is also known to be involved in chondrogenesis and cartilage regeneration.^[^ ^51^, ^54^ ^]^ We herein found that GDF5 induces anabolic factor expression, activin A regulates catabolic factor expression, and GDF8 and 11 do not affect anabolic or catabolic factor expression in our system. GDF5 did not share ACVR2B in its function of inducing anabolic factor expression, suggesting that it may bind to another receptor.^[^ ^55^ ^]^ Furthermore, the expression of activin A is increased by activin A, but not other ACVR2B ligands, and our GSEA demonstrated that an activin A‐specific gene signature contains genes that contribute to regulating the expression of catabolic factors in OA development but not chondrogenesis. The results of our in vitro experiments strongly suggest that activin A is specifically increased under OA pathogenic conditions and regulates the expression of catabolic factors, such as MMP3, MMP13, and COX‐2, through ACVR2B. Consistent with our in vitro results, our in vivo experiments revealed that activin A Tg mice experience enhanced osteoarthritic cartilage destruction via MMP3, MMP13, and COX‐2, whereas heterozygous activin A KO mice show blockade of OA development. Taken together, these findings indicate that activin A functions as a unique ACVR2B ligand and plays important roles in accelerating OA pathogenesis through catabolic factor expression.

We further characterized the downstream mechanism(s) through which the activin A‐ACVR2B axis mediates OA pathogenesis. From among the various OA pathogenic biochemical pathways, in silico and biochemical analyses revealed that activin A is strongly related to ROS production in Ad‐activin A‐infected chondrocytes. Endogenous ROS is generated through the NAD(P)H oxidase (NOX) system, and ROS overproduction regulates extracellular matrix degradation through catabolic factor expression.^[^ ^33^ ^]^ Interestingly, NOX4, which is a major modulator of ROS, is the most highly expressed NOX isoform during the development of OA.^[^ ^35^ ^]^ Since the expression patterns of activin A, ACVR2B, and NOX4 in human OA cartilage are essentially similar to those in OA cartilage of mice, the present study serves as a critical starting point for understanding ACVR2B‐mediated OA pathogenesis.

Of the various ROS production‐related genes, we found that Nox4 was highly upregulated in activin A‐overexpressed chondrocytes and activin A Tg mice, whereas knockdown or knockout of Nox4 inhibited activin A‐induced ROS production. Moreover, the overexpression of Nox4 upregulated MMP3, MMP13, and COX‐2 in chondrocytes and Nox4 KO mice exhibited decreased osteoarthritic cartilage destruction. Interestingly, NOX4 consists of six TM domains: Its N‐ and C‐terminal moieties both face the cytosol,^[^ ^36^ ^]^ and its C‐terminus was predicted to interact with a C‐terminal binding site on ACVR2B. Our protein structural homology modeling and immunoprecipitation assays also revealed that the C‐terminal sequence of ACVR2B directly interacts with Nox4. Moreover, we found that DMM‐operated activin A Tg:Nox4 KO mice were protected against osteoarthritic cartilage destruction.

We next characterized the signaling pathway triggered by the activin A‐ACVR2B‐Nox4 axis in chondrocytes. Once activin A binds ACVR2B, this axis should recruit a type I receptor (ACVR1A, ACVR1B, ACVR1C, BMPR1A, or BMPR1B), which is essential for Smad signaling activation.^[^ ^26^, ^56^ ^]^ Interestingly, *ACVR1B^−/−^* mice do not survive beyond embryonic day 9.5 because the egg cylinder undergoes developmental arrest before gastrulation.^[^ ^57^ ^]^ However, pSmad2/3 expression was reportedly decreased in the skin epidermis and hair follicle epithelia of adult ACVR1B ^flox/flox^; K14‐Cre knockout (KO) mice.^[^ ^58^ ^]^ The Smad signaling pattern of these conditional ACVR1B KO mice was an exact match for that previously reported in a study of in vitro ACVR1B‐dependent Smad signaling.^[^ ^59^ ^]^

Although SB431542 and LDN193189 are known as inhibitors of ACVR1B and ACVR1, respectively, SB431542 also inhibit ALK4, 5, and 7^[^ ^60^ ^]^ and LDN193189 potently inhibits ALK1, 2, 3, and 6.^[^ ^61^ ^]^ Thus, it would be difficult to use these inhibitors to clearly characterize the type I receptors in OA pathogenesis. To more specifically characterize the involvement of ACVR1B in catabolic factor expression, we performed experiments using siRNA against the type I receptors, ACVR1A, ACVR1B, ACVR1C, BMPR1A, and BMPR1B. Our results showed that activin A regulates catabolic factor expression through Smad2/3 phosphorylation via ACVR1B, but not the other type I receptors.

Our co‐IP and in silico structure modeling analyses further demonstrated direct interactions of activin A‐ACVR2B‐Nox4 in vitro. Previous reports suggested that an interaction of the Smad2/3 complex with AP‐1 could regulate target gene expression in the nucleus.^[^ ^40^ ^]^ Here, we sought to define a transcription factor related to the activin A‐mediated stimulation of articular chondrocytes. Among the examined transcription factors, overexpression of activin A or Nox4 most highly upregulated the AP‐1 transcription factor complex component, Fosl1. rActivin A treatment or Ad‐activin A infection increased the expression and activity of HIF‐2*α*, which has an AP‐1 binding site in its promoter. Conversely, an AP‐1 inhibitor blocked HIF‐2*α* expression in chondrocytes. These findings suggest that AP‐1‐induced HIF‐2*α* and ACVR1B are required to complete the ACVR2B assembly for inducing OA through the Smad2/3 signaling pathway. Although blockade of the ability of the Fosl1‐mediated AP‐1 complex to regulate HIF‐2*α* was found to inhibit catabolic factor expression in our system, pathogenic ligands are generally considered to be better targets for the therapeutic blockade, compared to transcription factors. We thus propose a new therapeutic paradigm for directly trapping ACVR2B ligands with sACVR2B‐Fc to address OA pathogenesis. ACVR2A‐Fc, which has been tried in clinical trials, should mitigate the progression of OA because it could have a ligand specificity similar to that of ACVR2B‐Fc.^[^ ^62^ ^]^ However, we herein demonstrate that ACVR2A is not expressed or involved in OA progression, and a previous report suggested that ACVR2B can bind activin A with a higher affinity than shown for another of its ligands.^[^ ^44^ ^]^ Indeed, GDF5, 8, 11, which are known as ligands of ACVR2A and ACVR2B,^[^ ^63^ ^]^ did not regulate catabolic factor expression or appear to be involved in OA progression. These data collectively suggested that it may be difficult to use sACVR2A‐Fc in treating OA. Furthermore, our data indicate that trapping activin A as an ACVR2B ligand can protect against DMM and metabolic‐induced OA pathogeneses. From our collective findings, we conclude that the pathogenic ACVR2B assembly could affect various types of arthritis pathogenesis. The blockade of ACVR2B or its ligands may be a useful strategy for disrupting the completed ACVR2B assembly, offering opportunities to understand the mechanisms of and develop new therapeutic approaches against OA.

## 4 Experimental Section

### Human OA Cartilage and Experimental OA in Mice

Human OA cartilage was sourced from individuals undergoing arthroplasty (Table S3, Supporting Information). All patients provided written informed consent, and the collection was approved (UC14CNSI0150). All animal experiments were ethically approved by the Animal Care and Use Committee of Ajou University School of Medicine (IACUC 2016‐0041). To perform mouse experiment and estimate to calculate in vivo experiments, previous publications were followed.^[^ ^31^, ^64^ ^]^ C57BL/6J mice were used for the experimental OA studies. C57BL/6J‐background activin A heterozygous KO mice (*Inhba* ^+/−^) and homozygous Nox4 KO mice (*Nox4* ^−/−^) were purchased from Jackson Laboratory. Because *Inhba* ^−/−^ mice are embryonic lethal,^[^ ^32^ ^]^ *Inhba* ^+/−^ mice were used for the experimental OA study. Cartilage‐specific activin A Tg mice (Col2a1‐*Inhba*) were generated using the Col2a1 promoter and enhancer (Macrogen, Seoul, Korea), as described previously.^[^ ^31^ ^]^ Spontaneous cartilage‐specific activin A Tg mice were used for histopathological analysis at 18 months of age. Experimental OA was induced in 12 week old male mice by DMM surgery. Mice were treated as indicated and subjected to histological and biochemical analyses. For the metabolism‐related OA mouse model, the high‐fat diet (HFD) model^[^ ^45^ ^]^ was used in which 10‐week‐old mice were fed either a normal chow diet (ND) or HFD. DMM surgery was performed in the same week mice were first exposed to the ND or HFD. Mice were treated as indicated and subjected to histological and biochemical analyses.

### Intraarticular (IA) Injection in Mice

Adenoviruses encoding control shRNA (Ad‐shC) or shRNA against ACVR2B (Ad‐shACVR2B) were purchased from Vector Biolabs (Malvern, PA, USA) and IA was injected into mouse knee joints as described previously.^[^ ^64^ ^]^ After the onset of DMM‐induced OA, Ad‐shACVR2B was administrated once per week via IA injection. sACVR2B‐Fc was purchased from Y Biologics (Daejeon, Korea) and injected into the knee joints of mice at 4 weeks after DMM surgery (10 µg in a total volume of 10 µL). Control mice received Fc‐control (10 µg in a total volume of 10 µL; Y Biologics).

### Histological and Immunohistochemical Analyses

Human OA cartilage was sectioned (10 µm thickness) and fixed in 4% paraformaldehyde. Cartilage sections were stained with Alcian blue to detect sulfate proteoglycans. Mouse knee joints were excised and fixed in 4% paraformaldehyde, decalcified for 2 weeks in 0.5 m EDTA, and embedded in paraffin. Paraffin blocks were serially sectioned (5 µm thickness), and the sections were deparaffinized in xylene, hydrated through graded ethanol solutions, and stained with Safranin‐O. Cartilage destruction was assessed by three observers who were blinded to the experimental grouping, and scored according to the OARSI grading system (grade 0–6). OARSI scores are presented as the mean maximum score for each mouse. Each representative Safranin‐O staining image was selected from the most advanced lesion among the serial sections. ACVR2B, activin A, MMP3, MMP13, COX‐2, pSmad1/5, pSmad2/3, Gdf5, Gdf8, and Gdf11 were detected by immunohistochemical staining of human and mouse cartilage sections with the following antibodies: anti‐MMP3 (Abcam, Cambridge, UK), anti‐MMP13 (Abcam, Cambridge, UK), anti‐activin A (Proteintech, Rosemont, IL, USA), anti‐ACVR2A (Santa Cruz Biotechnology, Dallas, Texas, USA), anti‐ACVR2B (Thermo Fisher Scientific, Waltham, MA, USA), anti‐pSmad1/5, anti‐pSmad2/3 (Cell Signaling Technology, Danvers, MA, USA), anti‐Gdf5 (Abcam, Cambridge, UK), anti‐Gdf8 (Proteintech, Rosemont, IL, USA), and anti‐Gdf11 (Thermo Fisher Scientific, Waltham, MA, USA). All signals were quantified using the ImageJ software v1.60.

### Microcomputed Tomography (µCT) Image Acquisition and Analysis

The samples were scanned using a SkyScan 1173 (Bruker, Kontich, Belgium) using the key scan parameters: applied source voltage, 90 kVp; applied source current, 88 µA; isotropic image voxel size, 10 µm; exposure, 500 ms; frame average, 4; number of projections, 659; rotation step, 0.3 degree; 180 degree scan. The bone mineral density phantom set was scanned under the same conditions used for the sample scans. After scanning, the acquired projection datasets were reconstructed in NRecon (Bruker, Kontich, Belgium) using an A1 1.0 mm filter and the key reconstruction parameters: ring artifact correction, 7; beam hardening correction, 40%; and attenuation coefficient dynamic range, 0–0.034. The reconstructed cross‐section sample datasets were reoriented in DataViewer (Bruker, Kontich, Belgium) for fine analysis, and the reoriented datasets were analyzed using CTAn (Bruker, Kontich, Belgium). A volume of interest (VOI) consisting of 100 slices starting from ≈0.5 mm proximal to the growth plate and spanning 1.6 mm in length was chosen for analysis. The VOI for the subchondral trabecular bone was contoured at a threshold corresponding to 30% of the maximum image gray scale; the entire load‐bearing VOI on the medial side^[^ ^65^, ^66^ ^]^ was used to calculate morphometric parameters, including the trabecular bone volume (BV/TV), trabecular thickness (Tb.Th), trabecular separation (Tb.Sp), trabecular number (Tb.N), cortical thickness (Ct.Th), and cortical tissue mineral density (Ct.TMD). The bone object was segmented by global thresholding (81‐255), not dynamic thresholding. The object mask was then refined by custom processing using the Despeckle plug‐in of BRUKER (Billerica, MA, USA). The Despeckle process (BRUKER, Billerica, MA, USA) may be similar to the low‐pass filtering process applied by the Scanco software (SCANCO Medical AG, Brüttisellen, Switzerland). After analysis, the visual output data for the 3D analyses of WT, Tg, KO, and DMM‐induced OA model mice were loaded to CTvox (Bruker, Kontich, Belgium) and processed for visual representation of the trabecular thickness distribution.

### Bioindentation

Bioindentation tests were performed with an Anton Paar Bioindenter (Anton Paar, Graz, Austria) as previously described^[^ ^67^ ^]^ using freshly dissected femoral condyle cartilage. The indentation was performed using a ruby ball indenter (200 µm). For each joint, at least three locations were tested on the load‐bearing region of the medial condyle to account for spatial heterogeneity. Fresh joint samples were kept in PBS (pH = 7.4) with protease inhibitors (Roche Holding AG, Basel, Swiss) at 4 °C for less than 24 h prior to testing. The effective indentation modulus (*E*_ind_) was calculated by fitting each force‐indentation depth‐loading curve with the Hertz model.

### Primary Cell Isolation, Culture, and Biochemical Treatments

Articular chondrocytes were isolated from cartilage tissues of 5 day old WT or activin A Tg mice. Each cartilage tissue sample was subjected to consecutive enzymatic digestions with proteinase and collagenase, as previously described.^[^ ^68^ ^]^ Adenoviruses expressing mouse activin A (Ad‐activin A) and Nox4 (Ad‐Nox4) and control empty adenoviruses (Ad‐C) were purchased from Vector Biolabs (Malvern, PA, USA). Chondrocytes were cultured for 2 d and infected with adenovirus for 2 h at the indicated multiplicity of infection (MOI).^[^ ^69^ ^]^ The postnatal chondrocytes from activin A Tg and WT littermates were grown for 24 h in serum‐free medium to 90% confluency. The supernatant medium was concentrated using Microsep Advance with 10K Omega (PALL, NY, USA) and treated as indicated. Chondrocytes were also treated with the indicated concentrations of recombinant IL‐1*β*, IL‐6, TNF‐*α*, IL‐17, IL‐21, GDF5, GDF8, GDF11, or activin A for the indicated periods. IL‐1*β*, IL‐6, TNF‐*α*, IL‐17, and IL‐21 were purchased from GenScript Biotech (Piscataway, NJ, USA). The following were purchased as indicated: Gdf5, Gdf8, Gdf11 (ProSpec, Rehovot, Israel), and activin A (R&D Systems, Minneapolis, MN, USA). Nontargeting (scrambled) siRNA (control) and siRNA specific to ACVR2A, ACVR2B, activin A, or Nox4 were purchased from Dharmacon (Lafayette, CO, USA). siRNA for ACVR1A, ACVR1B, ACVR1C, BMPR1A, and BMPR1B were purchased from Thermo Fisher Scientific (Waltham, MA, USA). Each siRNA was transfected into cells using RNAiMAX (Invitrogen, Waltham, MA, USA). Chondrocytes were transfected with siRNA for 6 h, and then adenoviruses were applied.

### Micromass Culture

Mesenchymal cells were isolated from the limb buds of E11.5 embryos and digested with trypsin and collagenase.^[^ ^70^, ^71^ ^]^ Cells (2 × 10^7^ cells mL^−1^) were spotted (15 µL per spot) on culture dishes for a 5 d induction of chondrogenesis. At 2 h post‐spotting, the dish was loaded with a medium containing 10 × 10^−3^ m *β*‐glycerophosphate (Sigma‐Aldrich, Saint Louis, Missouri, USA) and 50 µg mL^−1^ ascorbic acid (Sigma‐Aldrich, Saint Louis, Missouri, USA) for chondrogenic differentiation. To confirm that chondrogenesis was induced as intended, the expression patterns of Sox9, ColIIB, and aggrecan on day 1, 3, and 5 were monitored.^[^ ^72^ ^]^ To assess whether activin A could down‐ or up‐regulate chondrogenesis, mesenchymal cells with activin A (R&D Systems, Minneapolis, MN, USA) were treated on days 5 of micromass culture and examined the enrichment of ECM by Alcian blue staining. For Alcian blue staining, the cells were fixed with 4% paraformaldehyde for 10 min, stained with 1% Alcian blue solution for 30 min, and washed three times.

### Microarray Analysis, Ingenuity Pathway Analysis (IPA), and Gene Set Enrichment Analysis (GSEA)

To identify novel pathogenic receptors associated with OA pathogenesis, the GEO databases were used for human OA cartilage samples (GSE16464) and rat OA cartilage samples (GSE8077) and performed IPA (Qiagen, Venlo, Netherlands Hilden, Germany) and UniProtKB/Swiss‐Prot program‐based analyses. To screen for expression of ACVR2B ligands (GDF5, GDF8, GDF11, and activin A), microarray data were used from human OA cartilage (GSE16464), spontaneous OA cartilage of STR/ort mice, cartilage from CBA control mice (GSE33754), DMM‐induced OA cartilage (GSE26475), and IL‐1*β*‐treated human chondrocytes (GSE75181). Microarray analysis was also performed using chondrocytes infected with Ad‐activin A or Ad‐Nox4 or treated for 36 h with 200 ng mL^−1^ of recombinant GDF5, GDF8, GDF11, or activin A. Briefly, mouse articular chondrocytes were infected with 800 MOI of Ad‐activin A or empty virus (Ad‐C) for 36 h. Total RNA was isolated using the TRIzol reagent and analyzed using an Affymetrix GeneChip array (Affymetrix Mouse Gene 2.0 ST Array,  Santa Clara County, California, USA) and the Affymetrix protocol (Macrogen, Seoul, Korea). Microarray data were deposited in the Gene Expression Omnibus under accession codes GSE146271 (for activin A) and GSE146272 (for NOX4). The expression profiling results are available in a public repository of the NCBI SRA database (https://www.ncbi.nlm.nih.gov/sra) under the following accession numbers: GDF5 (SRR19347634), GDF8 (SRR19347633), GDF11 (SRR19347632), and activin A (SRR19347631). To identify genes associated with NOX4 and AP‐1, a list of genes differentially expressed in Ad‐activin A‐ or Ad‐Nox4‐infected chondrocytes was uploaded to the IPA software. Genes related to ROS production or transcriptional regulation were sorted, and analyzed gene profiles using GSEA and IPA. GSEA was carried out using the Broad Institute JAVA Desktop software (ver. 4.3) (www.broadinstitute.org/gsea),^[^ ^73^ ^]^ which applies nonparametric Kolmogorov‐Smirnov statistics to calculate whether the members of a given gene set show significant differences compared to the controls. The normalized enrichment score (NES) was determined by investigating 10 000 permutations. *p* < 0.05 was considered to signify statistically significant enrichment of a gene set. GSEA was performed using the ratio of class.

### Protein Structural Homology Modeling

The crystal structure of ACVR2B (accession ID: NP_001097.2, and PDB ID: 2QLU) was retrieved from the RCSB protein databank (https://www.rcsb.org). Homology‐based structural modeling of ACVR1B (accession ID: NP_0 04293.1) and NOX4 (accession ID: NP_05 8627.2) was performed using the SWISS‐MODEL web server (http://swissmodel.expasy.org) and AlphaFold2.^[^ ^74^, ^75^ ^]^ Computational docking simulations of ACVR1B, ACVR2B, and NOX4 were conducted with AlphaFold2 and ClusPro 2.0.^[^ ^75^, ^76^ ^]^ The graphical representation of docking structures was constructed using PyMOL (ver. 1.3; DeLano Scientific, Shirley, NY, USA).

### Collagenase Activity, PGE_2_ Assays, and Reporter Gene Assay

Chondrocytes were seeded to six‐well dishes (2 × 10^5^ cells per well) for 24 h and transfected with targeted or control siRNA. Cells were exposed to IL‐1*β* (1 ng mL^−1^) and incubated for an additional 24 h in Dulbecco's modified Eagle's medium (DMEM, Thermo Fisher Scientific, Waltham, MA, USA) without fetal bovine serum (FBS, Thermo Fisher Scientific, Waltham, MA, USA). The culture medium was collected and equal volumes were concentrated using Viva spin columns (Sartorius Stedim Biotech, Gottingen, Germany). Total collagenase activity in the concentrated samples was assayed using EnzChek Gelatinase/Collagenase Assay kits (Molecular Probes, Eugene, OR, USA). Collagenase activity was measured as a fluorescent signal using a VICTOR X3 microplate reader (PerkinElmer; Ex/Em = 495/515 nm). Production of PGE_2_ was assessed using a PGE_2_ immunoassay kit (R&D Systems, Minneapolis, MN, USA). Briefly, chondrocytes were seeded to six‐well plates (2 × 10^5^ cells per well), and the amounts of secreted and cellular PGE_2_ were quantified from total cell lysates by measuring absorbance at 560 nm. For reporter gene assays, the Hif‐2*α* reporter gene construct (GeneCopoeia, Rockville, MD, USA) was transfected into chondrocytes using LipofectAMINE Plus (Invitrogen, Waltham, MA, USA), as previously described.^[^ ^31^ ^]^ The transfected cells were cultured in complete medium for 24 h, and the Gaussia luciferase (GLuc) activity was determined using an assay kit (Abcam, Cambridge, UK) and normalized to the *β*‐galactosidase activity.

### RT‐PCR and qRT‐PCR

Total RNA was isolated from articular chondrocytes using the TRIzol reagent (Molecular Research Center, Cincinnati, OH, USA). cDNA was obtained by reverse transcription using ImProm‐II Reverse Transcriptase (Promega, Madison, WI, USA). WT, heterozygous activin A KO (*Inhba* ^+/−^), activin A Tg (Col2a1‐*Inhba*), and Nox4 KO (*Nox4* ^−/−^) mice were identified by polymerase chain reaction (PCR) of genomic DNA. The utilized PCR primers and experimental conditions are summarized in Table S4 (Supporting Information). PCR amplification was carried out using SYBR premix Ex Taq (TaKaRa Bio Inc., Shiga, Japan). For each target gene, the transcript levels were normalized to those of the GAPDH mRNA and expressed as the fold‐change relative to the indicated control.

### Immunoprecipitation and Western Blot Analysis

For immunoprecipitation, ACVR2B cDNA (NM_001106.4) was synthesized, and mutants of the putative Nox4 binding sites were generated using a site‐directed mutagenesis kit (iNtRON Biotech, Seongnam‐si, Korea) and subcloned into the p3XFLAG‐CMV‐7.1 plasmid (BioD, Gwangmyeong‐si, Korea). PolyJet (SignaGen Laboratories, Shandong Province, China) was used to transfect chondrocytes with control vector (p3XFLAG‐CMV‐7.1), p3XFLAG‐CMV‐7.1‐ACVR2B, or p3XFLAG‐CMV‐7.1‐muACVR2B (Mu1, with G373 changed to K373; Mu2, with G399 changed to K399; Mu3, with Q401 changed to K401; and Mu4, with G373, G399, and Q401 changed to K373, K399, and K401, respectively) for 36 h. Interaction of the endogenous ACVR2B‐ACVR1B heterodimer with Nox4 was assessed in IL‐1*β*‐treated or Ad‐activin A‐infected chondrocytes. Cellular proteins of each group were extracted with lysis buffer (150 × 10^−3^ m NaCl, 1% NP‐40, 50 × 10^−3^ m Tris, 0.2% sodium dodecyl sulfate (SDS), 5 × 10^−3^ m NaF) supplemented with a protease and phosphatase inhibitor cocktail (Roche Holding AG, Basel, Swiss). Proteins were incubated overnight at 4 °C with anti‐FLAG (Sigma‐Aldrich, St. Louis, MO, USA) or anti‐ACVR2B (Abcam, Cambridge, UK). The bound proteins were collected by incubation for 6 h at 4 °C with protein A/G plus beads (Roche Holding AG, Basel, Swiss), and washed with immunoprecipitation buffer. Precipitated proteins were separated by SDS‐PAGE and analyzed with antibodies against FLAG, ACVR1B, ACVR2B, or NOX4. For Western blotting, secreted MMP3 and MMP13 proteins were collected from a serum‐free conditioned medium by trichloroacetic acid (TCA) precipitation. Precipitated or cellular proteins were separated by SDS‐PAGE, transferred to a nitrocellulose membrane, and detected using anti‐ACVR2B (Thermo Fisher Scientific, Waltham, MA, USA), anti‐activin A (Santa Cruz Biotechnology, Dallas, TX, USA), anti‐Mmp3 (Abcam, Cambridge, UK), anti‐Mmp13 (Abcam, Cambridge, UK), anti‐Cox‐2 (Abcam, Cambridge, UK), anti‐Nox4 (ProteinTech Group, Rosemont, IL, USA), anti‐Erk1/2, pErk, Smad2/3, pSmad2/3, Smad1, pSmad1/5,p38, pp38, and ppJNK (Cell Signaling Technology, Beverly, MA). For the signaling pathway, the same membrane was stripped with Stripping Buffer (Thermo Fisher Scientific, Waltham, MA, USA). Protein bands were visualized using a SuperSignal West Dura kit (Thermo Fisher Scientific, Waltham, MA, USA). Band intensities were quantified by densitometric analysis (Alpha Ease FC 4.0; Alpha Innotech).

### Detection of ROS

For ROS analysis, chondrocytes isolated from postnatal WT and activin A Tg mice, infected with Ad‐activin A, Ad‐Nox4, or Ad‐C, or transfected with Nox4 siRNA and then infected with the indicated MOIs of Ad‐activin A were used. DPI (diphenyleneiodonium; Sigma‐Aldrich, St. Louis, MO, USA) was added 1 h prior to infection as a positive control. To detect intracellular ROS, chondrocytes were loaded with 20 × 10^−6^ m of 2’,7’‐dichlorofluorescin diacetate (DCF‐DA; Invitrogen, Waltham, MA, USA) and incubated for 30 min at 37 °C in the dark. After being washed and harvested, cells were immediately examined by flow cytometry using a FACS instrument (Miltenyi Biotec, Seoul, Korea) and analyzed with the FlowJo software (BD Bioscience, San Diego, CA, USA). Results are expressed as the mean fluorescence intensity. For oxidative stress analysis of mouse cartilage sections, immunohistochemical staining was performed with 8‐hydroxy‐2’‐deoxyguanosine (8‐OhdG; GTX41980, GeneTex, Irvine, CA, USA).

### Statistical Analysis

All experiments were performed independently at least five times (*n* = 5). Statistical comparisons were performed by first using the Shapiro‐Wilk test for normality or Levene's test for homogeneity of variance. For parametric data comparison, two groups were analyzed using the two‐tailed independent t‐test, while three or more groups were analyzed using one‐way analysis of variance (ANOVA) with Bonferroni's post‐hoc test. For non‐parametric data, two groups were analyzed using the Mann‐Whitney U test and multi‐groups were analyzed using Kruskal‐Wallis test or Friedman test followed by the Mann‐Whitney U test. Values are presented as mean ± SD, and a *p*‐value < 0.05 was considered statistically significant.

## Conflict of Interest

The authors declare no conflict of interest.

## Author Contributions

J.J. designed and conducted most of the in vitro and in vivo experiments and wrote the manuscript. H.L., S.E., and M.S.J. carried out computational analyses of microarrays, GEO, and protein structural homology modeling. S.J.K. obtained and evaluated human joint samples. C.C., K.W.K., and D.J.Y. conducted the µCT analysis. S.L., Y.S.B., W.I.C., and J. J. conducted experiments related to sACVR2B‐Fc, and Ad‐shAcvr2b injection analysis. S.E. (as co‐corresponding author) and S.Y. conceived, planned, and oversaw the study.

## Supporting information

Supporting Information

## Data Availability

The data that support the findings of this study are available in the supplementary material of this article.
