# Supplementary material for: Blockade of Activin Receptor IIB Protects Arthritis Pathogenesis by Non‐Amplification of Activin A‐ACVR2B‐NOX4 Axis Pathway
Source: Adv Sci (Weinh). 2023 Mar 22;10(14):2205161. doi: 10.1002/advs.202205161 (PMC10190289; doi:10.1002/advs.202205161)
Supplement: Supplementary file 1 — Supporting Information [file ADVS-10-2205161-s001.pdf]

ADVANCED SCIENCE
Open Access

Supporting Information

for *Adv. Sci.*, DOI 10.1002/advs.202205161

Blockade of Activin Receptor IIB Protects Arthritis Pathogenesis by Non-Amplification of Activin A-ACVR2B-NOX4 Axis Pathway

Jimin Jeon, Hyemi Lee, Min-Seung Jeon, Seok-Jung Kim, Cham Choi, Ki Woo Kim, Dong Joo Yang, Sangho Lee, Yong-Soo Bae, Won Il Choi, Juyeon Jung, Seong-il Eyun and Siyoung Yang**

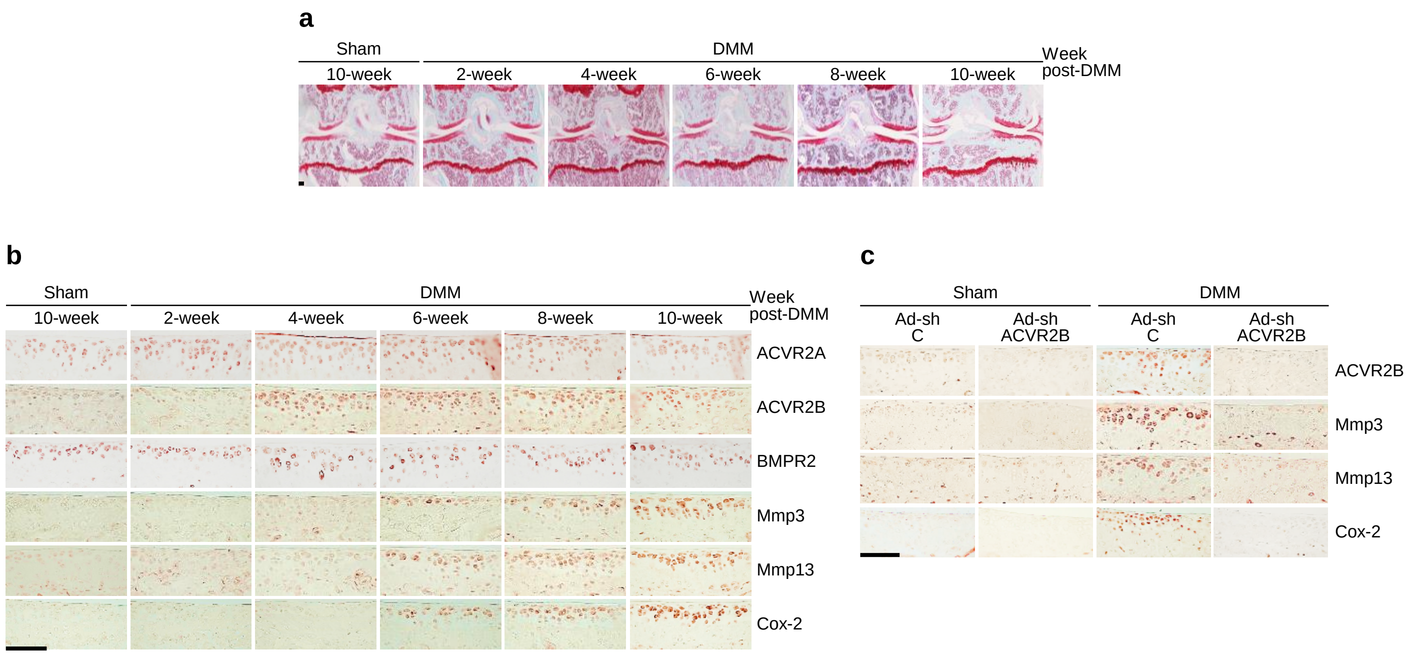

Figure S1. The expression levels and effects of ACVR2B in the OA mouse models. a,b) Operated mice were sacrificed at the indicated weeks after surgery (*n* = 5). Representative Safranin-O staining images of cartilage sections (a) and immunostaining images (b). c) Representative immunostaining images for ACVR2B, Mmp3, Mmp13, and Cox-2 in cartilage. Scale bar: 100 µm.

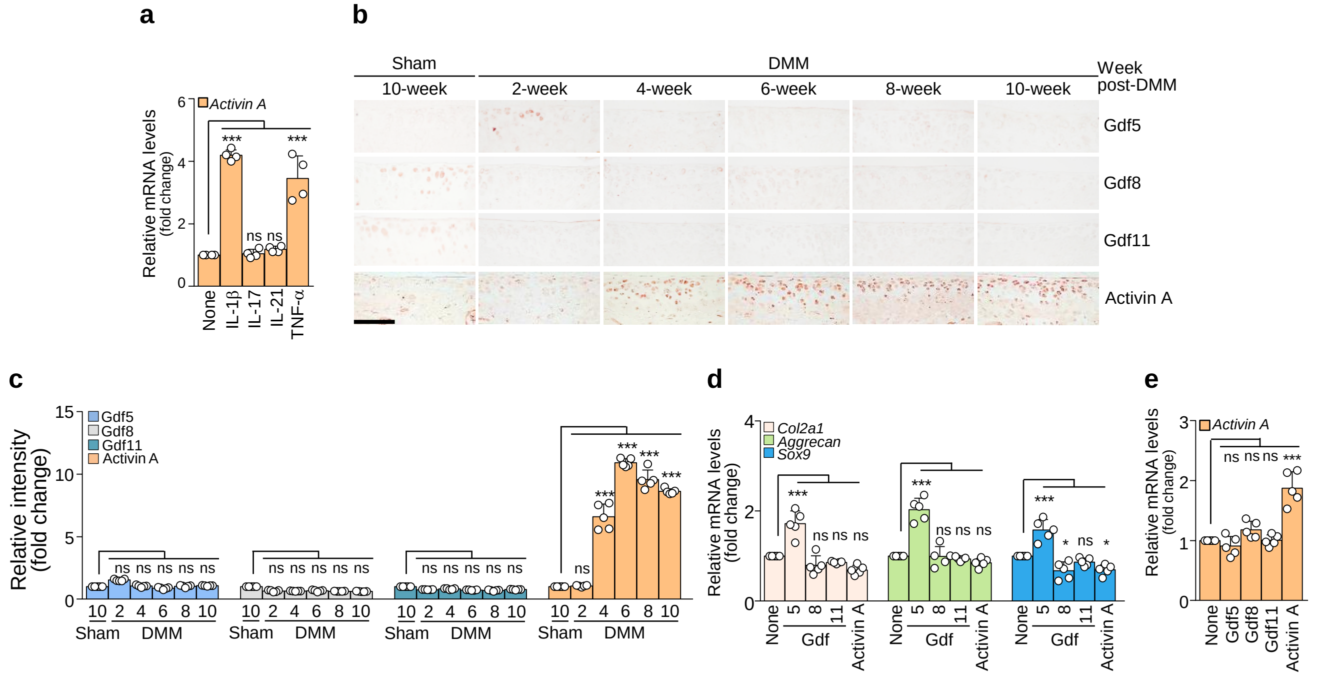

Figure S2. The expression levels of Gdf5, 8, 11, and activin A in OA pathogenesis. a) The relative mRNA levels of activin A were determined by qRT-PCR analysis (n = 4) in primary-culture mouse chondrocytes treated for 36 h with IL-1β (1 ng/ml), IL-17 (50 ng/ml), IL-21 (100 ng/ml), or TNF-α (50 ng/ml). b,c) Representative immunostaining images (b) and immunostaining intensity (c) of DMM-operated mice sacrificed at the indicated weeks after surgery (n = 5). d) The relative mRNA levels of *Col2a1*, *aggrecan*, and *Sox9* were determined by qRT-PCR analysis (n = 5) in chondrocytes treated with rGdf5 (200 ng/ml), rGdf8 (200 ng/ml), rGdf11 (200 ng/ml), or rActivin A (200 ng/ml). e) Activin A expression was determined by qRT-PCR analysis of chondrocytes treated with Gdf5, 8, 11, or activin A (n = 5). Values are presented as means ± SD and were assessed using one-way ANOVA with Bonferroni's *post-hoc* test (a,c,d,e). *p < 0.05; **p < 0.01; ***p < 0.001, ns; not significant. Scale bar: 100 μm.

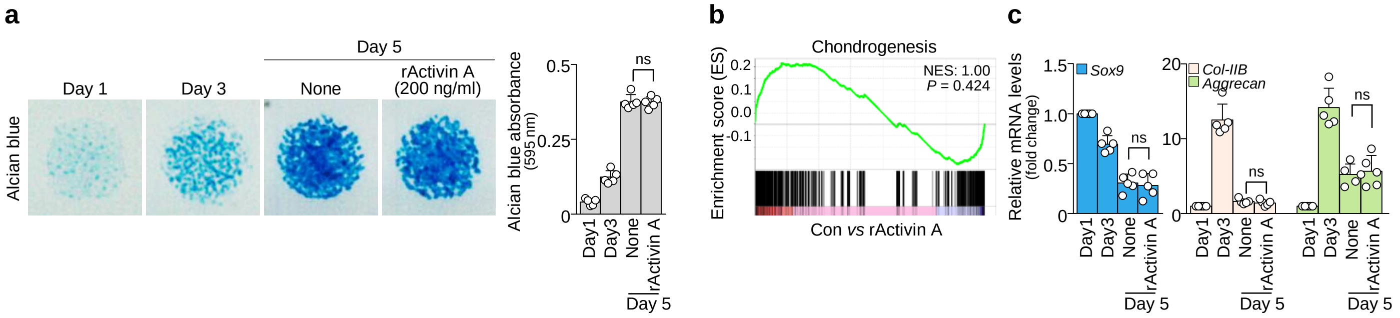

Figure S3. Activin A does not affect chondrogenesis. a) Representative Alcian blue staining images (*left*) and absorbances (*right*). Accumulation of sulfated proteoglycans was determined by Alcian blue staining (*n* = 5). b) GSEA of chondrogenesis in chondrocytes treated with 200 ng/ml of recombinant activin A. Normalized enrichment score (NES) and nominal p-value are indicated. c) qRT-PCR results of the indicated molecules in mesenchymal cells treated with activin A on 5 day of micromass culture (*n* = 5). Values are presented as means ± SD and were assessed using two-tailed *t*-test (a,c). ns, not significant.

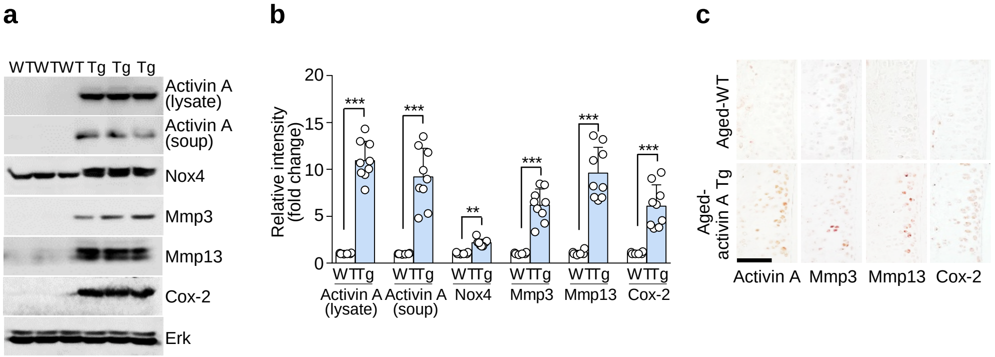

Figure S4. Cartilage-specific activin A Tg mice exhibit induced catabolic factor expression. a,b) Representative Western blot images (a) and relative protein intensity levels (b) of the indicated molecules in primary-culture chondrocytes of littermate activin A Tg mice (n = 9) and WT mice (n = 5). c) Immunostaining images of the indicated molecules in cartilage sections of 18-month-old activin A Tg mice. Values are presented as means ± SD and were assessed using two-tailed *t-test* (b). **p < 0.01; ***p < 0.001. Scale bar: 100 μm.

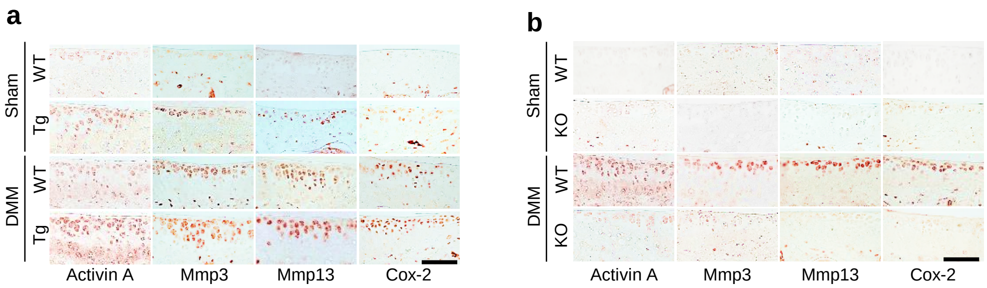

Figure S5. Activin A regulates catabolic factor expression. a) Representative immunostaining images of the indicated molecules in cartilage of sham- or DMM-operated WT and activin A Tg mice (*n* = 10). b) Representative immunostaining images of the indicated molecules in cartilage of sham- or DMM-operated WT and KO mice (*n* = 10). Scale bar: 100 μm.

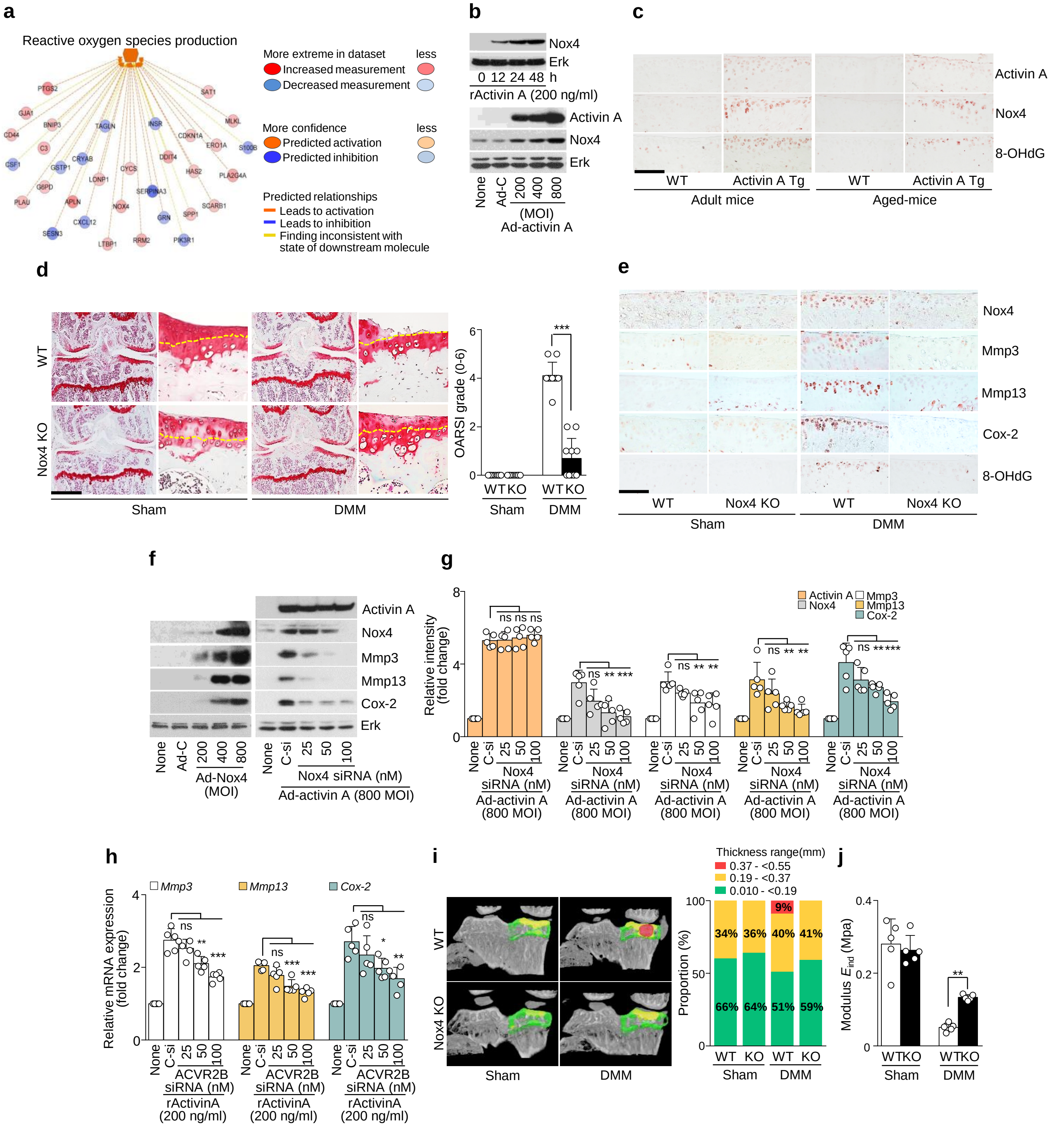

Figure S6. Activin A regulates ROS production through the Nox4 pathway in chondrocytes. a) IPA results of ROS-related genes in chondrocytes infected with 800 MOI of Ad-C or Ad-activin A for 36 h. b) Representative Western blot images of the indicated molecules in chondrocytes treated with recombinant activin A (rActivin A) for 48 h or infected with Ad-C (800 MOI) or Ad-activin A for 36 h (*n* = 5). c) Representative immunostaining images of the indicated molecules in cartilage sections obtained from activin A Tg littermate mice at 10 weeks old (*left*) and 18 months old (*right*). d) Representative Safranin-O staining images (*left*; yellow dotted lines indicate tidemarks) and scoring of OARSI grade (*right*) (*n* = 10). e) Immunostaining images of the indicated molecules in cartilage of DMM-induced WT and Nox4 KO mice. f,g) Representative Western blot images (f) and relative protein intensity levels (g) of the indicated molecules in chondrocytes. The chondrocytes were infected with the indicated MOI of Ad-Nox4, Ad-activin A, or Ad-C (800 MOI) for 36 h in the absence or presence of 100 nM of control siRNA (C-si) or the indicated concentrations of Nox4 siRNA (*n* = 5). h) Expression levels of *Mmp3, Mmp13*, and *Cox-2* in rActivin A-treated chondrocytes further treated with the indicated concentrations of ACVR2B siRNA (*n* = 5). i) Representative reconstructed 3D µCT images of posttraumatic OA-induced WT and Nox4 KO mice (*left*) and stacked-bar plot showing the trabecular bone thickness distribution in the indicated samples (*right*) (*n* = 5). j) The elastic modulus of cartilage, as measured by bioindentation (*n* = 5). Values are presented as means ± SD and were assessed using Kruskal-Wallis test followed by Mann-Whitney U test (d,j), or one way ANOVA with Bonferroni's *post-hoc* test (g,h). $^*p < 0.05$; $^{**}p < 0.01$; $^{***}p < 0.001$, ns; not significant. Scale bar:100 µm.

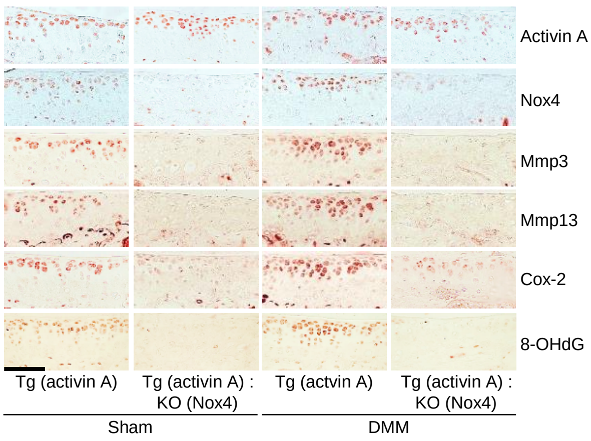

Figure S7. Nox4 is a catabolic mediator of the activin A-ACVR2B axis in OA pathogenesis. Representative immunostaining images of the indicated molecules in cartilage sections of sham- or DMM-operated activin A Tg mice and activin A Tg:Nox4 KO mice (*n* = 10). Scale bar: 100 μm.

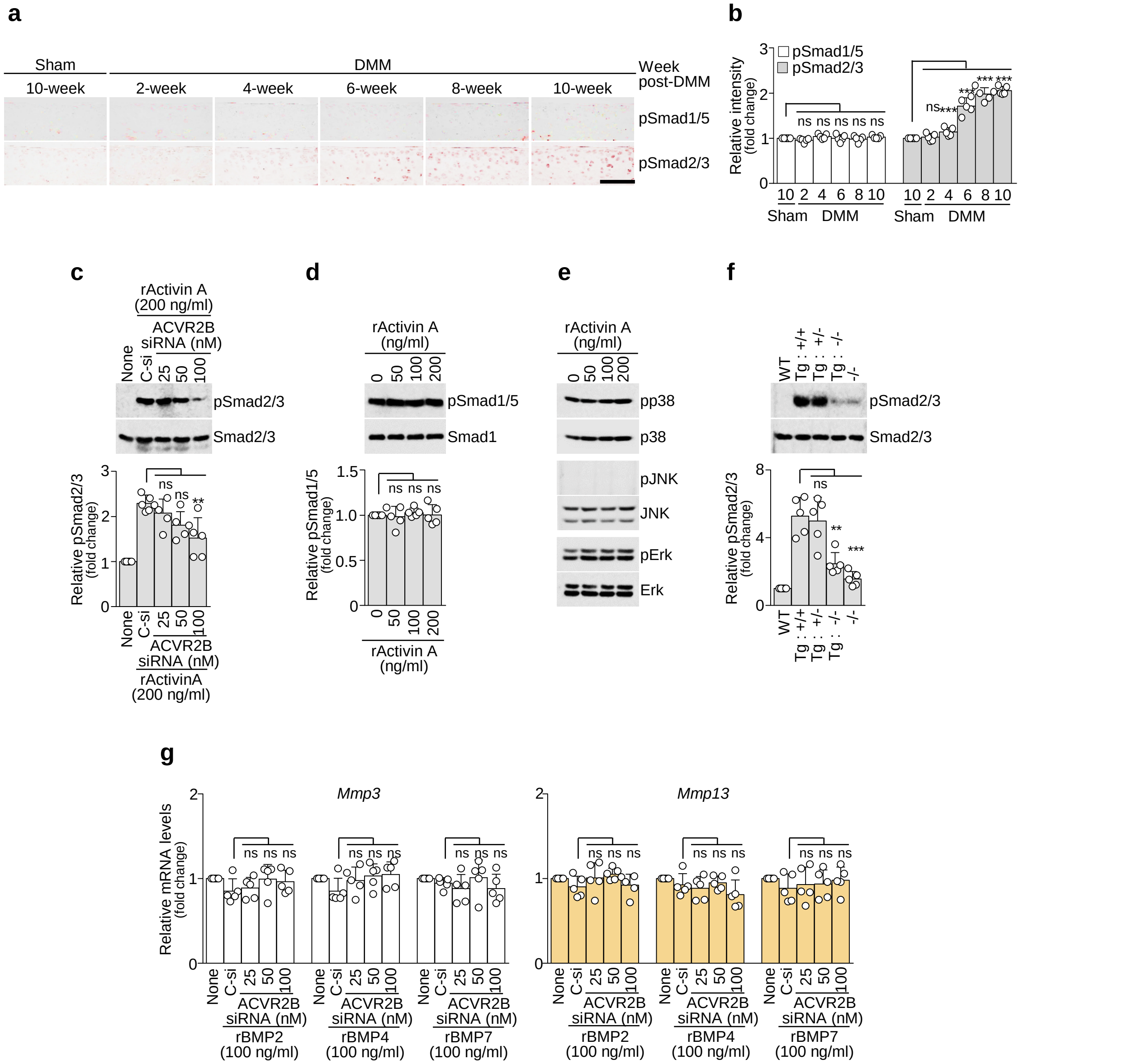

Figure S8. The activin A-ACVR2B-Nox4 axis regulates catabolic factor expression through Smad2/3 phosphorylation, but not Smad1/5 phosphorylation or non-Smad signaling. a,b) Representative immunostaining images (a) and immunostaining intensity (b) of DMM-operated mice sacrificed at the indicated weeks after surgery (*n* = 5). c) Representative Western blot images (c, *upper*), and image density (c, *lower*) of ractivin A treated chondrocyte in in the absence or presence of ACVR2B siRNA (*n* = 5). d) Representative Western blot images of the indicated molecules in rActivin A-treated chondrocytes (*upper*) and relative protein intensity levels (*lower*) (*n* = 5). e) Representative Western blot images of proteins related to the non-Smad signaling pathway in rActivin A-treated chondrocytes. f) Representative Western blot images of the indicated molecules in chondrocytes of WT, activin A Tg;Nox4 KO, and Nox4 KO mice (*upper*) and relative protein intensity levels (*lower*) (*n* = 5). g) Relative mRNA levels of *Mmp3* and *Mmp13* were determined by qRT-PCR analysis (*n* = 5) in chondrocytes treated with rBMP2, 4, or 7 plus the indicated concentrations of ACVR2B siRNA. Smad2/3, Smad1, p38, Erk, and JNK used for loading control. Values are presented as mean ± SD and were analyzed using one-way ANOVA with Bonferroni's *post-hoc* test (b,c,d,f,g). **p < 0.01; ***p < 0.001. ns, not significant. Scale bar: 100 μm.

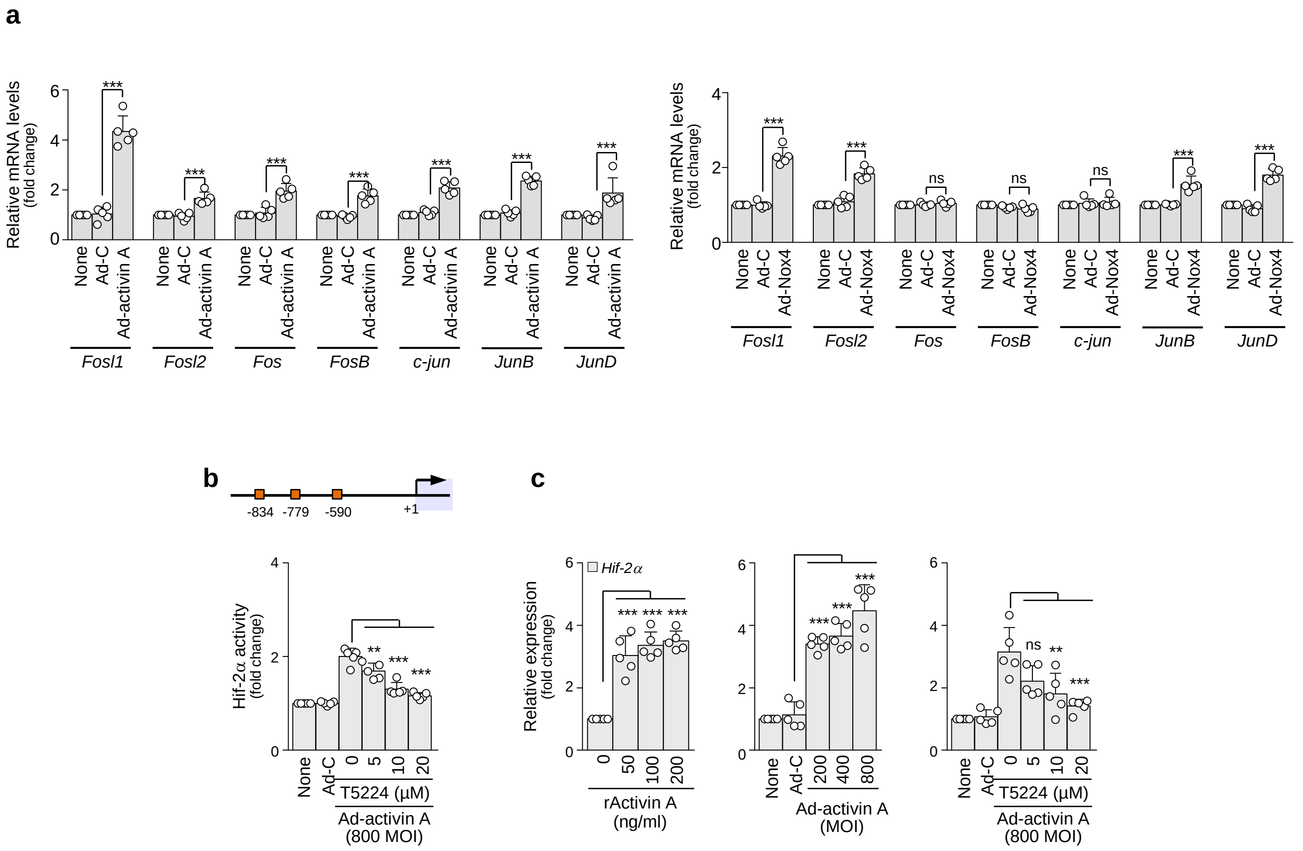

Figure S9. The AP-1 complex binds to the Hif-2α promoter and regulates Hif-2α expression. a) Relative mRNA levels of AP-1 complex components (*Fosl1, Fosl2, Fos, FoxB, C-jun, JunB, and JunD*) were determined by qRT-PCR analysis in Ad-activin A or Ad-Nox4-infected chondrocytes, compared to Ad-C-infected chondrocytes (n = 5). b) Diagram of the AP-1 binding site in the Hif-2α promoter (*upper*). Chondrocytes transfected with Hif-2α reporter gene constructs were infected with Ad-activin A in the absence or presence of T5224. The Hif-2α activity was determined by luciferase assay (n = 5) (*lower*). c) Relative mRNA levels of *Hif-2α* were determined by qRT-PCR analysis (n = 5) in primary chondrocytes treated with activin A (200 ng/ml), infected with 800 MOI of Ad-C or Ad-activin A, or treated with the indicated concentrations of T5224 and Ad-activin A. Values are expressed as mean ± SD and were assessed using one-way ANOVA with Bonferroni's *post-hoc* test (a,b,c). **p < 0.01; ***p < 0.001, ns; not significant.

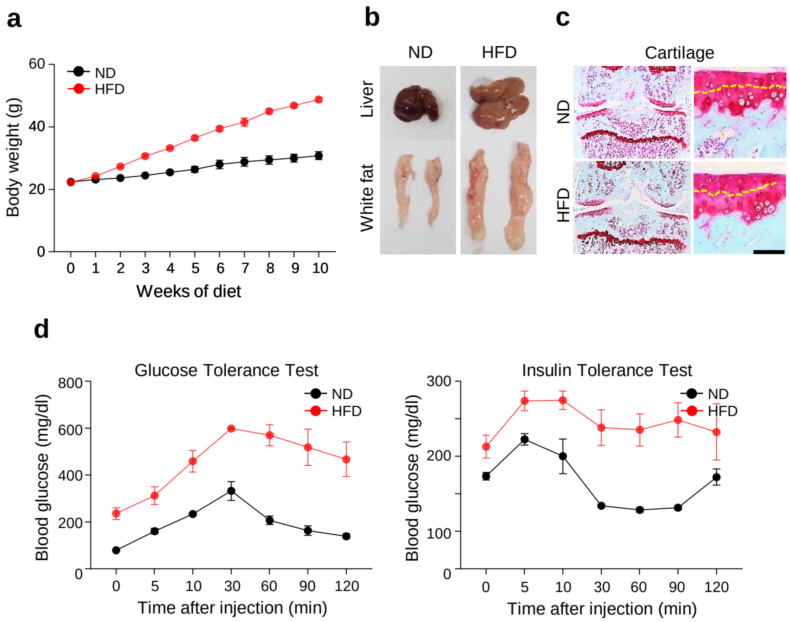

Figure S10. Characterization of high-fat diet (HFD)-fed mice. a) Body weights of normal chow diet (ND; black line, *n* = 5) and HFD (red line, *n* = 5)-fed mice were monitored over 10 weeks. b,c) Representative images of liver (b, *upper*), white fat (b, *lower*), and knee-joint cartilage (c). d) Glucose tolerance (*left)* and insulin tolerance (*right)* test data from the ND and HFD groups. Tidemarks are indicated by yellow dotted lines (c). Values are presented as means ± SD and were assessed by Friedman test followed by Mann-Whitney U test (a,d). Scale bar: 100 μm.

Table S1. Top 50 gene products related to single-pass transmembrane (TM) receptors in OA-related GEO datasets.

	Human OA patients (GSE16464)		OA rat cartilage (GSE8077)	
	Gene product	Fold change	Gene product	Fold change
Single-pass TM proteins	AMIGO2	22.51	ACVR2B	7.69
	SORCS3	17.00	PIGR	5.01
	LRIT1	16.16	LRRC15	4.86
	RPRM	13.81	F3	4.85
	ACVR2B	10.64	CD40	4.43
	CLCA4	9.10	PTPRB	4.38
	SCARF1	7.52	PODXL2	4.24
	IGSF11	7.43	NOTCH2	4.10
	ZP2	7.30	TNFRSF1B	4.06
	DPP10	6.35	AREG	3.92
	LAMP3	5.19	LILRA5	3.85
	IL2RG	4.42	NRXN1	3.37
	RNF128	4.39	CUZD1	3.24
	TP53I13	4.33	TGFA	3.20
	QSOX2	4.03	FAT4	2.99
	PRSS8	3.91	CDHR1	2.80
	CUZD1	3.68	FCGR2B	2.78
	CD3D	3.67	LRTM2	2.64
	RPRML	3.64	HBEGF	2.61
	ITGAM	3.47	PLB1	2.59
	LRRC4B	3.18	ADAM23	2.57
	SELP	3.18	LRRC4C	2.49
	EFNB1	3.03	TNFRSF9	2.49
	IMPG2	3.00	TMEM132A	2.46
	PCDHB9	2.97	MUC4	2.46
	FGFR3	2.64	MRC1	2.46
	LINGO1	2.60	PVR	2.45
	SIGLEC5	2.54	L1CAM	2.42
	ITGAD	2.54	STAB1	2.40
	LRP4	2.48	EMCN	2.39
	SPN	2.47	ANPEP	2.38
	IL7R	2.39	MMP14	2.34
	SEMA4B	2.35	TEK	2.32
	CD40LG	2.27	PECAM1	2.31
	IGSF9	2.27	FLT4	2.31
	SHISA9	2.24	ITGAX	2.30
	PCDH8	2.24	TRHDE	2.30
	TRAT1	2.22	CD79B	2.21
	TNFRSF19	2.22	CD6	2.20
	LINGO2	2.15	CD2	2.16
	VSIG10	2.10	IL17RC	2.15
	PKHD1L1	2.09	DSG2	2.13
	LYVE1	2.07	TNFSF11	2.12
	AMHR2	2.03	CLEC14A	2.12
	KEL	2.01	SHISA9	2.11
	SDK1	2.00	FLT3	2.10
	PMEL	1.98	SEZ6	2.08
	ICOS	1.98	PTGFRN	2.08
	SELE	1.97	RPRM	2.08
	TRIL	1.91	IL6R	2.05

Table S2. The Activin A-specific gene signature, compared to those for GDF5, 8, and 11.

No	Gene product	Fold change	No	Gene product	Fold change
1	Nos2	1138.13	41	Fabp5	6.839304
2	Mmp3	718.2645	42	Gpr35	6.80139
3	Cxcl3	572.865	43	Nox4	6.368869
4	Saa2	367.357	44	Runx1	6.255352
5	Saa1	193.006	45	Sox10	6.00211
6	Lcn2	189.38	46	Cd44	5.995196
7	Chil1	60.04524	47	Sod2	5.936947
8	Il13ra2	51.3991	48	Ptx3	5.864973
9	Tnf	47.8981	49	Mt1	5.313853
10	Bdkrb1	47.2223	50	H2-K1	5.003272
11	Slc39a8	43.57009	51	Lrrc32	4.997257
12	Gbp5	42.5462	52	Serpina3n	4.977058
13	Slpi	42.33811	53	Vcan	4.902311
14	Mmp13	41.97903			
15	Camp	41.5781			
16	Rnd1	30.62297			
17	Nfkbiz	25.96223			
18	Cck	24.7549			
19	Tnip3	24.7174			
20	Icam1	24.02359			
21	Lbp	22.87653			
22	Cebpd	17.45323			
23	Serpinb2	16.0685			
24	Tlr2	14.67313			
25	Ptgs2	14.64753			
26	Tm4sf1	14.55205			
27	Gbp4	13.5919			
28	Junb	13.46341			
29	Sod3	12.80706			
30	Mt2	12.64216			
31	Tnfaip2	12.10723			
32	Nfkbia	11.64182			
33	Ier3	11.49411			
34	H2-T10	9.5626			
35	Fbln2	9.117141			
36	Slco1a6	8.76539			
37	Fas	8.761132			
38	Vnn1	7.802018			
39	Gbp2b	7.06123			
40	Tnnt2	7.01148			

Table S3. Characteristics of the human specimens used in this study.

No	Age (years)/ gender	ICRS grade	Joint	Height (m)	Weight (Kg)	BMI (kg/ m^2)	Use
1	65/F	4	Knee	158	53	21.23	IHC
2	80/F	4	Knee	143.3	55.1	26.83	IHC
3	72/F	4	Knee	165	65	23.88	IHC
4	63/F	4	Knee	152	52	22.51	IHC
5	69/F	4	Knee	151	60	26.31	IHC
6	73/F	4	Knee	153.8	70.75	29.89	IHC
7	63/F	4	Knee	156	72.2	29.67	IHC
8	73/F	4	Knee	154	83	35	IHC
9	75/F	4	Knee	154	55	23.19	IHC
10	63/F	4	Knee	163	74.3	27.96	IHC

Table S4. Primer sequences and qRT-PCR conditions.

[a]S, sense primer; As, antisense primer. [b]AT, annealing temperature.

Gene product	Origin	Strand[a]	Sequence	Size (bp)
ACVR2B	Mouse	S	5'-GGCTGCGTTTGGAAAGCTC-3'	133
		As	5'-GCAACAAGTTTTCGTGCTTCA-3'	
Activin A	Mouse	S	5'-TGAGAGGATTTCTGTTGGCAAG-3’	207
		As	5'-TGACATCGGGTCTCTTCTTCA-3'	
Nox4	Mouse	S	5'-TTTCTCAGGTGTGCATGTAGC-3'	201
		As	5'-GCGTAGGTAGAAGCTGTAACCA-3'	
Mmp3	Mouse	S	5'-CTGTGTGTGGTTGTGTGCTCATCCTAC-3'	350
		As	5'-GGCAAATCCGGTGTATAATTCACAATC-3'	
Mmp13	Mouse	S	5'-TGATGGACCTTCTGGTCTTCTGGC-3'	473
		As	5'-CATCCACATGGTTGGGAAGTTCTG-3'	
Col2a1	Mouse	S	5'-CACACTGGTAAGTGGGGCAAGA-3'	173
		As	5'-GGATTGTGTTGTTTCAGGGTTCG-3'	
Aggrecan	Mouse	S	5'-GAAGACGACATCACCATCCAG-3'	581
		As	5'-CTGTCTTTGTCACCCACACAT-3'	
Sox9	Mouse	S	5'-GAGCCGGATCTGAAGAGGGA-3'	153
		As	5'-GCTTGACGTGTGGCTTGTTC-3'	
Collagen-IIB	Mouse	S	5'-GGGTCTCCTGCCTCCTCCTGCTC-3'	204
		As	5'-TCCTTTCTGCCCCTTTGGCCCTAATTTTCGG-3'	
Gdf5	Mouse	S	5'-CCATCACACCCCACGAATACA-3'	215
		As	5'-CTTCCGTAAGATCCGCAGTTC-3'	
Gdf8	Mouse	S	5'-AGTGGATCTAAATGAGGGCAGT-3'	144
		As	5'-GTTTCCAGGCGCAGCTTAC-3'	
Gdf11	Mouse	S	5'-CTGCGCCTAGAGAGCATCAAG-3'	202
		As	5'-TCTCGGTGGTAGCGTGGTA-3'	
ACVR1A	Mouse	S	5'-TTTAAGAGACGCAATCAAGAGCG-3'	191
		As	5'-TCCAACAGGGTTATCTGGCGA-3'	
ACVR1B	Mouse	S	5'-CGTGTCTACCATAACCGCCAG-3'	189
		As	5'-GCCCTTGCCGATAATCTCTTGTA-3'	
ACVR1C	Mouse	S	5'-ATCGTAGGAAAAGGTCGGTTTG-3'	154
		As	5'-CGAGGATGTTCTCGTGTCTCA-3'	
ACVR2A	Mouse	S	5'-ATAAACGGCGACATTGTTTTGC-3'	234
		As	5'-TCGGTGTAACAGGATTTGAAGTG-3'	
BMPR2	Mouse	S	5'-TTGGGATAGGTGAGAGTCGAAT-3'	115
		As	5'-TGTTTCACAAGATTGATGTCCCC-3'	
Gapdh	Mouse	S	5'-TCACTGCCACCCAGAAGAC-3'	450
		As	5'-TGTAGGCCATGAGGTCCAC-3'	
Activin A	Activin A TG genotyping	S	5'-GGGCCCCTCTGCTAACCATGTT-3'	295
		As	5'-GGTCTCTTCTTCAAGTGCAGC-3'	
Activin A	Activin A KO genotyping	S (WT)	5'-GGGCTCAGAGATGAAGCAGT-3'	251
		S (Mu)	5'-AGACTGCCTTGGGAAAAGCG-3'	
		As	5'-TGGCCCTTTAAAACTCATGG-3'	110
Nox4	Nox4 KO genotyping	S	5'-GTGGTCCAACAG AACAACTGC-3'	228
		As (WT)	5'-CACAAGTCTCCTAGTCAAAAGTGA-3'	
		As (Mu)	5'-AACGTCGTGACTGGGAAAAC-3'	350
